# Optimisation of Retsina Wine Quality: Effects of Resin Concentration, Yeast Strain, and Oak Chip Type

**DOI:** 10.3390/foods13213376

**Published:** 2024-10-24

**Authors:** Pantelis I. Natskoulis, Dimitrios-Evangelos Miliordos, Apostolos N. Koutsouris, Petros A. Tarantilis, Christos S. Pappas, Stamatina Kallithraka, Yorgos Kotseridis, Maria Metafa

**Affiliations:** 1 Institute of Technology of Agricultural Products, Hellenic Agricultural Organization—DIMITRA, 1 Sofokli Venizelou, 141 23 Lycovrisi, Greece; p.natskoulis@elgo.gr (P.I.N.);; 2 Laboratory of Oenology and Alcoholic Drinks, Department of Food Science & Human Nutrition, Agricultural University of Athens, 75 Iera Odos, 118 55 Athens, Greece; toliskoutsouris@gmail.com (A.N.K.); stamatina@aua.gr (S.K.); ykotseridis@aua.gr (Y.K.); 3 Laboratory of Chemistry, Department of Food Science & Human Nutrition, Agricultural University of Athens, 75 Iera Odos, 118 55 Athens, Greece; ptara@aua.gr (P.A.T.);

**Keywords:** Savatiano, resinated wine, retsina, oak chips, oxidisability tests, volatile compounds, organic acids, phenolic compounds

## Abstract

Retsina, Greece’s most renowned traditional wine, has been produced for millennia, with archaeological and historical evidence supporting its legacy. It is legally defined as wine made exclusively in Greece using grape must infused with Aleppo pine resin (*Pinus halepensis*). This study examines the effects of varying resin concentrations (0.5 g/L and 1 g/L), two commercial yeast strains, and medium-toast oak (Nadalié Cooperage, Ludon-Médoc, France) American and French, on Retsina’s chemical and sensory properties to optimise its production. Wine samples from the Savatiano grape variety were analysed for classical wine parameters, oxidation stability, volatile compounds, organic acids, phenolic profiles, and sensory attributes. Principal Component Analysis (PCA) revealed that yeast strain selection significantly influences chemical composition, with Zymaflore X5 associated with higher organic acid levels. Oak addition altered phenolic profiles, with American oak increasing ellagic acid, while non-oaked wines showed higher syringic and p-coumaric acids. Resin addition elevated alpha-pinene, a key marker of resin aroma, but reduced esters linked to fruity and floral notes. These findings highlight the complex interactions between resin, yeast, and oak, offering insights for enhancing Retsina’s quality while preserving its traditional character.

## 1. Introduction

Greek resinated wine, more commonly known as “Retsina”, belongs to wines of Greece with Protected Geographical Indication (PGI), and additionally, it is protected by EU legislation as a product of Traditional Specialty Guaranteed (TSG), highlighting its unique production process and composition, as well as protecting it against falsification and misuse [1,2].

Retsina is a dry white or rose wine with a characteristic balsamic aroma of pine and an imperceptible bitterness, which leaves a refreshing aftertaste akin to carbonated refreshments, but at the same time allows the aesthesis of the flowery and fruity aroma of Savatiano and Roditis varieties [3,4]. Retsina, in contrast to the first impression of a rather moderate palate, with a common floral taste of white wines and hazelnut tones, is characterised by a very long and complex aftertaste, with butter-creamy, roasted peanut, and fir notes, usually met with wood-aged quality wines [5].

The primary distinction between resinated wine and classic vinification is the addition of up to 1% resin (oleoresin) from *Pinus halepensis* Mill. trees (*Aleppo pine*) at the beginning or during alcoholic fermentation (before the sugar content depletes to 2/3 of its initial concentration). Additionally, Retsina can bear the PGI designation if it is produced within the specified geographical area indicated on the label and complies with the relevant legal standards. According to Greek legislation, to qualify for the PGI designation, Retsina must be made primarily from Savatiano grapes or a blend with Rhoditis at a 10:1 ratio, with these varieties comprising at least 85% of the total grape content when other varieties are used [6,7].

The oldest evidence of resin application in vinification is reported by McGovern et al. [8] from a neolithic (c. 5400–5000 B.C.) wine jar excavated at Mount Zagros of North-West Iran, which proved to contain oleoresin residues of the *Pistacia atlantica* Desf. terebinth tree. A similar research study by this group supported further the use of pine resin as a natural wine preservative in jars recovered from shipwrecks in the Aegean Sea and coasts of western Anatolia and Corfu, dated from the 5th–3rd centuries B.C. [9], while Foley et al. presented DNA evidence of the common presence of *Pinus* and *Vitis* spp. in ancient Greek amphoras dating also between the 5th–3rd centuries B.C. [10]. Finally, a comprehensive review from Harutyunyan and Malfeito-Ferreira [11] gathered all relative historical documents to reveal a vinification timelapse among ancient civilisations of the Mediterranean basin, with those of epic Greek poet Homer (c. 8th century B.C.) and Greek historian Herodotus of Halicarnassus (c. 484–425 B.C.) being directly linked to ancient Greece heritage on winemaking. This heritage, and its survival through centuries to the present day under the technological process of Retsina, is also highlighted in the works of McKay et al. [12], Buglass [13], and Harutyunyan and Malfeito-Ferreira [14], all connecting the preservative and antioxidant properties of resin to Retsina’s prolonged shelf-life and specific aromatic character.

Resin is a complex mixture of terpenes, consisting of a volatile fraction of monoterpenes and sesquiterpenes, also known as turpentine, and a non-volatile fraction of diterpene resin acids known as rosin [15,16]. Pine resin is collected in Greece by pine-resin collectors and their associations, principally by the traditional tapping method of pine’s mechanical wounding and collection of the secreted resin in plastic or metal containers by gravity [17,18]. The most productive *Pinus* sp. in terms of resin secretion in Greece and the greater Mediterranean basin is *Pinus halepensis* Mill. (Aleppo pine), presenting an average yield of 2.7 kg per year, which may reach up to 25 kg or more for certain Greek Aleppo genotypes [19]. 

The chemical composition of the resin secreted by Greek Aleppo pine (*Pinus halepensis*) includes a diverse range of monoterpenes, which constitute approximately 30% of the resin. Among these, α-pinene is present in moderate amounts (about 20–35%) compared to other species, but it makes up roughly 90–95% of the total turpentine fraction. Other common monoterpenes include β-pinene, camphene, myrcene, δ-3-carene, and limonene. Sesquiterpenes, which account for up to 1.5% of the total resin, are primarily represented by β-copaene, sativene, longifolene, caryophyllene, and α-humulene. Diterpenes, which are the predominant constituents of the resin, can constitute up to 70% in some samples, with abietic, palustric, and levopimaric acids being the most abundant, followed by dehydroabietic, neoabietic, isopimaric, and pimaric acids in smaller amounts [18,19,20]. The composition of the resin is influenced by genetic factors and environmental conditions, while its quality and quantity are significantly affected by the tree’s age, origin, and environmental stresses such as drought, extreme temperatures, and flooding [20].

Savatiano (*Vitis vinifera* L.), the primary grape variety used for Retsina production, is the most widely cultivated white grape variety in Greece. It has been systematically cultivated in Attica, where it accounted for over 90% of grape vineyard area until recent years, although its prevalence is now decreasing. Savatiano is also commonly grown in other Greek regions, including Evia, Viotia, the Cyclades, Western Crete, the Peloponnese, and Macedonia [21]. This variety is known for its moderate vigour and high productivity. In older vineyards, it forms a steep or low cup with a trunk height of about 20 cm, while in younger vineyards, it is trained using a bilateral Royat system. Savatiano is adaptable to various soil types, but its quality is enhanced when grown in arid soils with adequate calcium levels and moderate fertility. Wines produced from Savatiano grapes are often described as “floral” and “citrus” with low acidity [22,23,24].

Traditionally, and before the modernisation of winemaking with stainless tanks, the fermentation of Retsina, as almost for every wine, was taking place in wooden oak barrels. Barrel-fermented Retsina is still a common practice. The aim of this work is to explore the potential for improving the quality of resinated wine by examining the effect of resin quantity, yeast strain, and presence of different types of oak chips, in single varietal Retsina made from the Savatiano cultivar. 

## 2. Materials and Methods

### 2.1. Vinification

#### 2.1.1. Must Preparation

Experimental microvinifications were conducted at the experimental winery of the Laboratory of Oenology and Alcoholic Drinks (L.E.A.D.) at the Agricultural University of Athens (A.U.A.), Athens, Greece. Grapes from *Vitis vinifera* cv. Savatiano, sourced from A.U.A. vineyards in the Spata region of Attiki (vintage 2017), were manually harvested and vinified. Upon arrival at L.E.A.D., the grapes were destemmed, crushed, and gently pressed using a hydraulic grape press with a pressure range of 0.5–0.7 bars. The grape juice underwent sulphitation with a concentration of 10 mg/L of sulphur dioxide (SO_2_), which was added during the crushing process. The pressed juice was then placed in a tank, and 3 mg/L of enzymes (Rohavin^®^ clear, AB Enzymes GmbH; Darmstadt, Germany) were added to facilitate sedimentation. Two hours later, 20 g/hL ScottLab^®^ Polycel (Scott Labs Ltd., Niagara-on-the-Lake, ON, Canada), 5 g/hL Tanin Antiox White, and 10 g/hL SpringArom^®^ (Fermentis, Lille, France) were added, and the must was left for static settlement at 10 °C for two days. After settling, the clear juice was racked off the sediment and further sulphited with 30 mg/L of sulphur dioxide (SO_2_).

#### 2.1.2. Yeast Strains

The clarified juice was divided into two tanks, each containing 135 L of must. Yeast inoculation followed using Saccharomyces cerevisiae active dry yeast strains: ZYMAFLORE^®^ X5 (Laffort S.A., Floirac, France) and VIVACE^®^ (Renaissance Yeasts Inc., Zug, Switzerland). The yeasts were rehydrated according to the suppliers’ recommendations and added to the must along with 25 g/hL of SpringFerm™ (Fermentis, Lille, France).

#### 2.1.3. Experimental Design and Applications of Resin and Wood Chips

Following yeast inoculation, the contents of the two fermentation tanks (ZYMAFLORE and VIVACE) were further divided into 54 containers of 5 L each (27 ZYMAFLORE and 27 VIVACE fermentation containers). Different concentrations of resin and types of wood chips were added to investigate their effects during vinification on the chemical composition and the sensorial descriptors of the produced wines. Three levels of resin application were tested (0 g/L, 0.5 g/L, and 1 g/L), and within each resin level, two types of wood chips (American and French) (1.5 g/L) were added. The wood chips used in the study were commercial products supplied by Oak Add-Ins Nadalié Cooperage (Ludon-Medoc), consisting of American oak (*Quercus alba*) and French oak (*Quercus robur*) [25]. On the 4th day of fermentation, diammonium phosphate (DAP) was added at a concentration of 25 mg/L. The resin was placed into the must enclosed in a gauze cloth bag, which was removed from the fermentation containers immediately after fermentation completion on the 13th day of vinification, while toasted oak chips were removed on the 30th day of vinification. Fermentations were conducted in a temperature-controlled room at 18–20 °C.

In total, 54 micro-vinifications were conducted per yeast treatment, organised according to a complete block design, with three repetitions for each factor. The experimental setup and design are illustrated in Figure S12. All micro-vinifications were carried out at the experimental winery of the Laboratory of Oenology and Alcoholic Drinks (LEAD).

### 2.2. Conventional Oenological Analyses

The conventional oenological parameters of retsina wines (pH, total acidity, volatile acidity, reducing sugars, free sulphur dioxide, colour intensity, and alcohol content) were determined according to the OIV protocols [26].

### 2.3. Total Phenolic Index and Follin–Ciocalteau

The total polyphenol index (TPI) of the wine samples was determined using a spectrophotometer set at a wavelength of 280 nm [27]. Prior to measurement, the wine samples underwent dilution. TPI was calculated by multiplying the absorbance value at 280 nm (A280 nm) by the dilution factor applied. For the determination of total phenolic content, spectrophotometric analysis was conducted at a wavelength of 760 nm, employing the Folin–Ciocalteu method [28]. Folin–Ciocalteu reagent (0.5 mL), diluted wine (1 mL), 20% *m*/*v* Na_2_CO_3_ solution (2 mL), and distilled water (2.4 mL) were sequentially added to the mixture. After a 30 min incubation period, absorbance was measured. A blank sample containing water instead of wine was used to correct background interference. All analyses were performed in triplicate to ensure the accuracy and reliability of the results. The results are expressed as mg/L gallic acid equivalents (GAE).

### 2.4. Antioxidant Activity

The determination of antioxidant activity was performed by employing the DPPH stable radical [29]. The results are expressed as mg/L Trolox equivalents (TE).

### 2.5. Accelerated Browning Test

The model utilised for evaluating the progression of browning underwent a modification based on the model initially outlined by Singleton and Kramling [30,31]. In this experimental setup, batches of wine consisting of 30 mL each underwent filtration before being carefully transferred into a 30 mL glass vial with a screw-cap lid, characterised by dimensions of 7.5 cm in length and an internal diameter of 2.1 cm. Subsequently, the wine samples were exposed to a consistent temperature of 55.0 ± 0.2 °C within a temperature-controlled water bath. At specific time intervals of 24 h spanning across a duration of 13 days, precise aliquots were extracted from the samples to evaluate the extent of browning, quantified by measuring the absorbance at 420 nm (A420). Following the measurement process, the samples were promptly reintroduced into their respective vials to ensure the preservation of the original headspace volume.

### 2.6. Determination of Volatile Compounds

#### 2.6.1. Sample Preparation

The extraction of volatile components from wine samples was conducted according to Ivanova et al. [32], with some modifications, as follows: 20 mL of the wine sample was spiked with 200 μL of internal standard solution (3-octanol). Subsequently, 8 mL of dichloromethane, used as the organic solvent for the extraction of volatile compounds, was added. The mixture was agitated using a vortex mixer for 1 min to ensure thorough mixing. Following this, the solutions were centrifuged (Z 36 HK High-Speed Tabletop Centrifuge, Hermle Labortechnik GmbH, Wehingen, Germany) for 15 min at 6000 rpm (3703× *g*), and the organic phase was carefully collected using a syringe. The collected organic phase was filtered to remove particulates, and approximately 300 mg of anhydrous sodium sulphate (Na_2_SO_4_) was added to absorb any residual aqueous phase.

The organic phase was then concentrated to a final volume of 1.5 mL under a stream of nitrogen gas and transferred into suitable vials for subsequent analysis.

#### 2.6.2. GC-MS Analysis

The analysis was performed using an Agilent 7890A Gas Chromatograph (GC) system, coupled with an Agilent 5975C VL Mass Selective Detector (MSD) with a Triple-Axis Detector (Agilent Technologies Inc., Santa Clara, CA, USA). The column used was a DB-WAX (30 m × 0.32 mm, 0.25 µm film thickness) from J & W Scientific (Folsom, CA, USA). Helium was used as the carrier gas at a flow rate of 1 mL/min. A 1 µL aliquot of the concentrated organic phase was injected into the system in split mode (1/50) at 220 °C. The oven temperature program started at 40 °C for 3 min, followed by an increase of 3 °C/min to 160 °C, and then an increase of 10 °C/min to 240 °C, where it was held constant for 10 min.

For the quantification, five-point calibration curves were generated for each analyte using simulated wine matrix calibration solutions containing a fixed concentration of 3-octanol as the internal standard. For the preparation of the simulated wine matrix calibration solutions, a synthetic wine model (12% ethanol and 4 g/L tartaric acid with pH 3.4, adjusted with sodium hydroxide solution) was prepared and spiked with appropriate volumes of standard solutions of the analytes and the internal standard. The liquid–liquid extraction (LLE) procedure, as previously described, was performed in triplicate for every concentration level. For the construction of the calibration curves, the relative peak areas of the analytes to the internal standard were used. 

The analytes in the samples were identified based on the retention time of the standards and by comparing the mass spectra of the peaks at the observed retention times with those of known compounds in the NIST library (https://webbook.nist.gov/chemistry/ (accessed on 20 February 2018)). This comparative analysis allowed for the accurate identification and quantification of the aromatic components present in the wine samples, ensuring the reliability and validity of the results obtained.

### 2.7. Organic Acid Analysis

The analysis was performed using a Waters 2695 Alliance liquid chromatograph system coupled with a Waters 2996 PDA detector (Milford, MA, USA), and utilised a Rezek ROA Organic Acid H+ (8%) column (7.8 × 300 mm) from Phenomenex (Torrance, CA, USA). Wine samples were prepared by centrifugation at 6000 rpm (3703× *g*) for 10 min, followed by filtration through a 0.2 μm syringe filter. A 20 μL volume of the filtered sample was injected into the chromatograph. The column compartment was maintained at 40 °C, with a flow rate of 0.5 mL/min and a total run time of 20 min. The mobile phase consisted of 5.0 mM H_2_SO_4_ in ultrapure water. Organic acids in the samples were detected at a wavelength of 210 nm. Compounds were identified based on the retention times of standards, and their quantification was performed using calibration curves derived from the peak areas of these standards.

### 2.8. Individual Phenolic Composition

The individual polyphenolic constituents were determined by HPLC. The analysis was performed using a Waters 2695 Alliance liquid chromatograph system coupled with a Waters 2996 PDA detector (Milford, MA, USA). Separation was performed on a reversed-phase Waters Nova-Pak C18 (150 × 3.9 mm, 4 μm) column with a 20 μL injection volume at a flow rate of 1 mL/min. Identification was achieved by comparing the retention times of the detected peaks with those of standard compounds, and by UV/VIS spectral data. All analyses were performed in triplicate. The chromatographic conditions are described in Kyraleou et al. [25].

### 2.9. Sensory Analysis

The sensory assessment was conducted by a panel of 16 trained and experienced individuals, aged 25 to 50 years, comprising master’s students, researchers, and professors from the LEAD group. Prior to participation, all panellists provided their consent. They attended two training sessions, where they were initially trained with standard solutions and subsequently served samples for assessment. The panellists identified the sensations and aromas perceived using a predetermined descriptor list. The selected attributes were grouped into three categories: visual descriptors (Colour Intensity), olfactory descriptors (Aroma Intensity, White Fruits, White Flowers, Vegetal/ Grassy Aroma, Resin Aroma, Vanilla Aroma, Spiciness, Woody Aroma), and gustative descriptors (Acidity, Bitterness, Astringency, Aftertaste). The tests were conducted in individual booths, with each sample served in a random order. Each panellist received 30 mL of wine in ISO wine glasses, served at room temperature (18–20 °C). Samples were presented with 3-digit blinding codes in a monadic sequence according to a Latin Square Design. The intensity of the sensory attributes was evaluated using a 5-point scale, where 0 indicated ‘null’ and 5 indicated ‘very strong’. The sensory evaluation took place in three sessions.

### 2.10. Statistical Analysis

Statistical analysis was conducted using GraphPad Prism (version 8.0.1, GraphPad Software). All data were subjected to a two-way analysis of variance (ANOVA) to determine the main and interaction effects of oak chip and resin treatments. For comparisons between means, the multiple Tukey range test was employed, with significance set at *p* < 0.05. Additionally, multivariate statistical data analysis (MVA) was performed using XLStat (XLSTAT 2017: Data Analysis and Statistical Solution for Microsoft Excel 2019; Addinsoft, Paris, France, 2017).

## 3. Results

### 3.1. Conventional Analysis of Must and Wine

Savatiano grapes were harvested at their optimum technological maturity in order to produce dry white wine, determined by measuring the grape berry Total Soluble Solids (TSS), Total Acidity, Density, pH, Yeast Assimilable Nitrogen (YAN), and the weight/berry. An evaluation of the grape must composition is presented in Table 1.

The effects of resin treatment (0 g/L, 0.5 g/L, and 1 g/L) and the type of oak chips (no oak, American oak, and French oak), including their interactions, were investigated in wines using a two-way ANOVA with Tukey’s HSD post hoc test (confidence limit at α = 0.05). The results of the conventional wine analysis are presented in Table 2.

In wines produced by both yeast strains, classic parameters such as alcoholic volume, reducing sugars, volatile acidity, total acidity, and SO_2_ were independent of the addition of resin and oak. Conversely, both yeast strains resulted in wines with significantly higher pH when no oak chips or resin were added. Additionally, it should be noted that all wines produced by the two commercial yeasts were dry and the acetic acid values were well below 0.6 g/L (Table 2).

### 3.2. Multivariate Statistics

The large dataset of 54 fermentations, characterised by 25 quantitative variables, offers the opportunity to investigate traits linked to the production of volatile, phenolic compounds, and organic acids. As a result, the effect of treatments (resin and oak chips) on different characteristics of a complex matrix, such as wines, cannot be fully understood using a conventional “one-at-a-time” approach. This conventional statistical method is limited in its ability to visualise the overall similarity between samples when considering all parameters simultaneously. Multivariate analysis, on the other hand, can identify similar interfaces within the experimental data by reducing many characteristics to fewer variables, thus facilitating interpretation. Principal Component Analysis (PCA) is a powerful multivariate analysis technique that recognises patterns within multidimensional data arrays. It allows for the identification of fewer inferences with similar characteristics, enabling a clearer understanding of the relationships and effects within the data.

**Figure 1:**
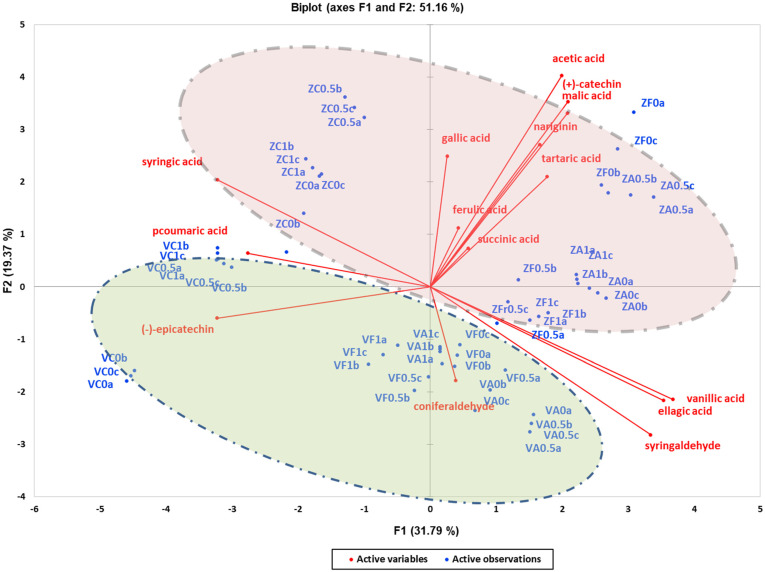
Unsupervised classification using principal component analysis on metabolomic data (phenolic compounds and organic acids) from the experimental wines produced by Vivace and Zymaflore X5 yeasts. The two circles highlight the two groups of wines, categorized by yeast strain. Letters of the samples in the Biplot indicate the ID: V—Vivace, Z—ZymafloreX5, F—French oak, A—American oak, C—control (no oak), 0—no resin, 0.5—0.5 g/L resin, 1—1 g/L resin, a; b; c—replication.

**Figure 2:**
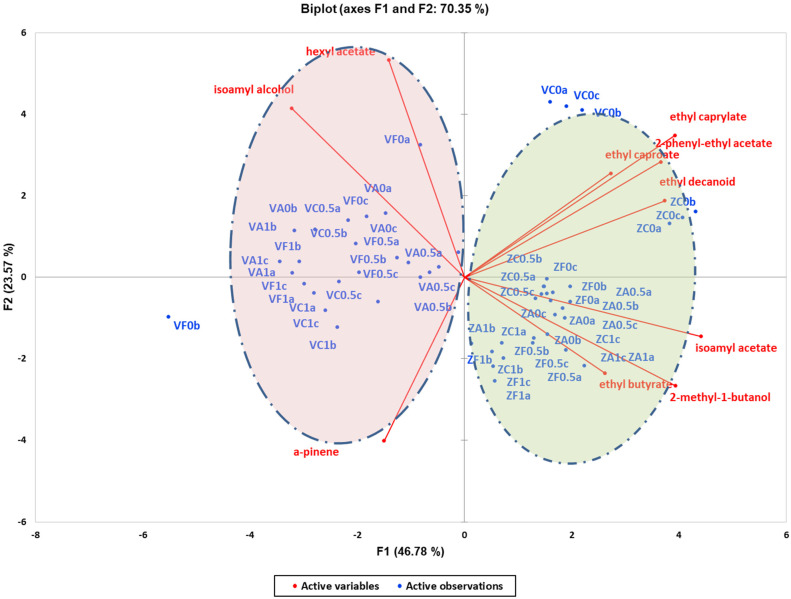
Unsupervised classification using principal component analysis on metabolomic data (volatile compounds) from the experimental wines produced by Vivace and Zymaflore X5 yeast strain. The two circles highlight the two groups of wines, categorized by yeast strain. Letters of the samples in the Biplot indicate the ID: V—Vivace, Z—ZymafloreX5, F—French oak, A—American oak, C—control (no oak), 0—no resin, 0.5—0.5 g/L resin, 1—1 g/L resin, a; b; c—replication.

To investigate the influence of two different yeast strains on volatile compounds, phenolic compounds, and organic acids, Principal Component Analysis (PCA) was conducted (Figure 1 and Figure 2). As shown in Figure 1, PC1 (31.79% variance explained) and PC2 (19.37% variance explained) effectively grouped the wines based on yeast strain. The PCA plot also reveals grouping between wines produced without oak additions (located on the left side) and those produced with oak wood additions (American and French chips, located on the right side). Organic acids such as tartaric, malic, acetic, and succinic were observed to scatter predominantly in the first quadrant, defined by positive values of PC1 and PC2. This suggests a positive correlation with wines produced by the Zymaflore X5 yeast strain. Regarding phenolic compounds, significant differences were noted within each yeast strain compared to wines without oak additions. Specifically, higher levels of vanillic acid, ellagic acid, syringaldehyde, and coniferaldehyde were observed, contributing to statistically significant increases in these compounds (Figures S5–S8). Conversely, wines without oak chips exhibited higher levels of syringic acid and *p*-coumaric acid. 

The PCA conducted on volatile compounds between the two different yeast strains with the addition of resin and oak chips (Figure 2) revealed similar trends to those observed in Figure 1. PC1 (46.78% variance explained) and PC2 (23.57% variance explained) effectively separated the treatments based on yeast strain. In Figure 2, wines produced without oak and resin are clustered together in the upper right part of the PCA plot. Conversely, wines produced by Zymaflore X5 yeast with resin and oak additions were clustered in the lower right part, while those produced by Vivace yeast were clustered on the left side of the PCA. Volatile compounds such as ethyl decanoate, ethyl caprylate, ethyl caproate, and 2-phenyl ethyl acetate were predominantly located in the first quadrant, characterised by positive values of PC1 and PC2. This indicates a positive correlation with wines produced by both yeast strains without resin and oak additions. Furthermore, most of the Vivace wine samples treated with resin and oak, clustered in the second quadrant (negative PC1, positive PC2), exhibited higher concentrations of hexyl acetate and isoamyl alcohol compared to Zymaflore X5 wine samples. In terms of volatile compounds, significant differences were noted within each yeast strain with resin and oak treatments compared to wines without these additions. Specifically, ethyl decanoate, ethyl caprylate, ethyl caproate, and 2-phenyl ethyl acetate showed statistically significantly higher concentrations, while wines treated with resin had elevated levels of α-pinene. Overall, the results are based on quantitative data. Figures S5–S8 highlight the statistically significant impact of the resin × oak interaction on the majority of targeted volatile and phenolic compounds in the wines.

**Figure 3:**
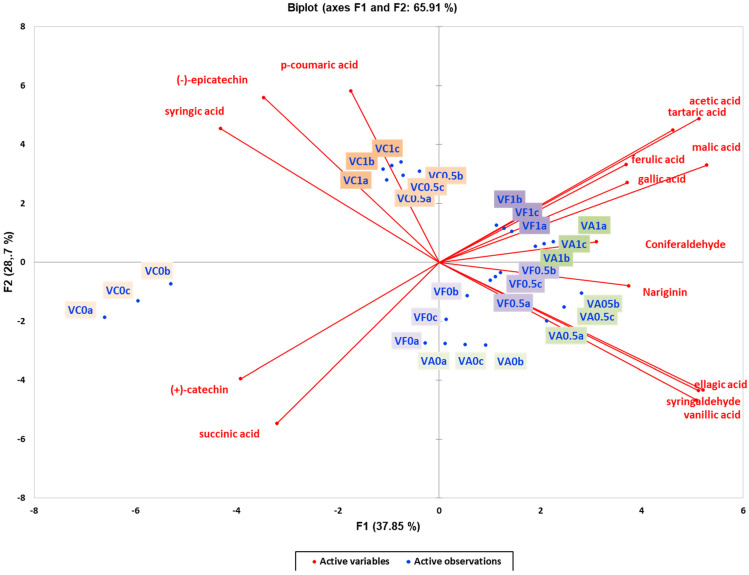
Unsupervised classification using principal component analysis on metabolomic data (phenolic compounds and organic acids) from the experimental wines produced by the Vivace yeast. Letters of the samples in the Biplot indicate the ID: V—Vivace, F—French oak, A—American oak, C—control (no oak), 0—no resin, 0.5—0.5 g/L resin, 1—1 g/L resin, a; b; c—replication. Samples in the score plots were coloured according to the resin treatments (0, 0.5, and 1 g/L) and oak treatment (no oak—orange, French oak—purple, American oak—green). Sequential increase in the resin concentration is presented with darker colours (0 g/L resin—pale, 0.5 g/L—medium dark, and 1 g/L—dark).

**Figure 4:**
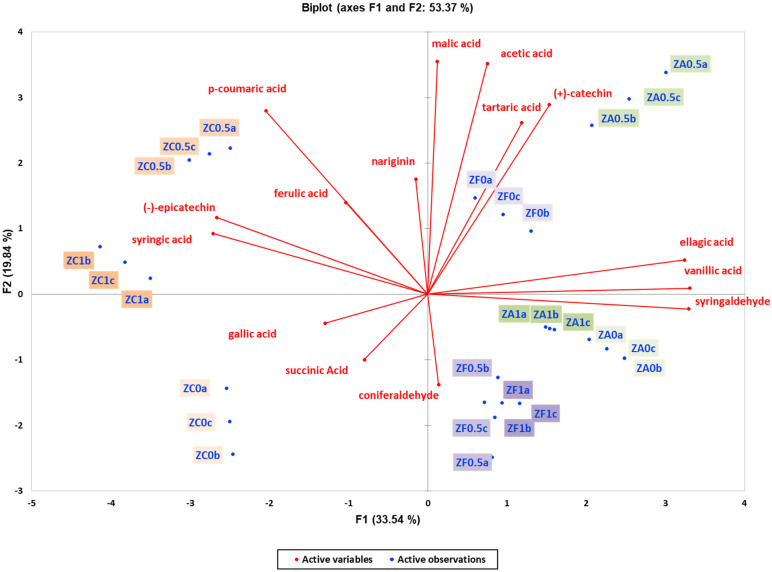
Unsupervised classification using principal component analysis on metabolomic data (phenolic compounds and organic acids) from the experimental wines produced by the ZYMAFLORE X5 yeast. Letters of the samples in the Biplot indicate the ID: Z—ZymafloreX5, F—French oak, A—American oak, C—control (no oak), 0—no resin, 0.5—0.5 g/L resin, 1—1 g/L resin, a; b; c—replication Samples in the score plots were coloured according to the resin treatments (0, 0.5 and 1 g/L) and oak treatment (no oak—orange, French oak—purple, American oak—green). Sequential increase in the resin concentration is presented with darker colours (0 g/L resin—pale, 0.5 g/L—medium dark, and 1 g/L—dark).

To investigate how different yeast strains impact wine quality, various subsets of metabolomic data (aroma compounds, phenolic compounds, and organic acids) were analysed using unsupervised methods. Principal Component Analysis (PCA) was employed to explore the changes in phenolic compounds and organic acids in the experimental wines. In experiments with Vivace and Zymaflore X5 yeasts, the PCA plots explained 65.91% (Figure 3) and 53.37% (Figure 4) of the total variance, respectively. Unlike volatile compounds, the primary factor that effectively grouped the wines was the effect of oak chips, with a clear classification between the two yeast strains according to the PC1 and PC2 axis of the analysis (Figure 3 and Figure 4). This indicates that combining all the presented parameters (phenolic compounds and organic acids) allows for successfully grouping and emphasising the effects of the two main factors (resin concentration and oak chips) on the wine samples. In both PCA biplots, wine samples without oak chips clustered on the left, while those with American and French oak chips clustered on the right (Figure 3 and Figure 4). The projection of ellagic acid, vanillic acid, and syringaldehyde indicated that these phenolic compounds effectively classified the wines, with a decreasing trend in wines without oak treatment (Figure 3 and Figure 4). The PCA results suggested that ellagic acid, syringaldehyde, and vanillic acid were found in higher concentrations in wines with oak addition, whereas wines with resin but no oak were characterised by *p*-coumaric, (-)-epicatechin, and syringic acid. Wines treated with neither resin nor oak were characterised by the presence of the organic acid succinic acid (Figure 3 and Figure 4).

**Figure 5:**
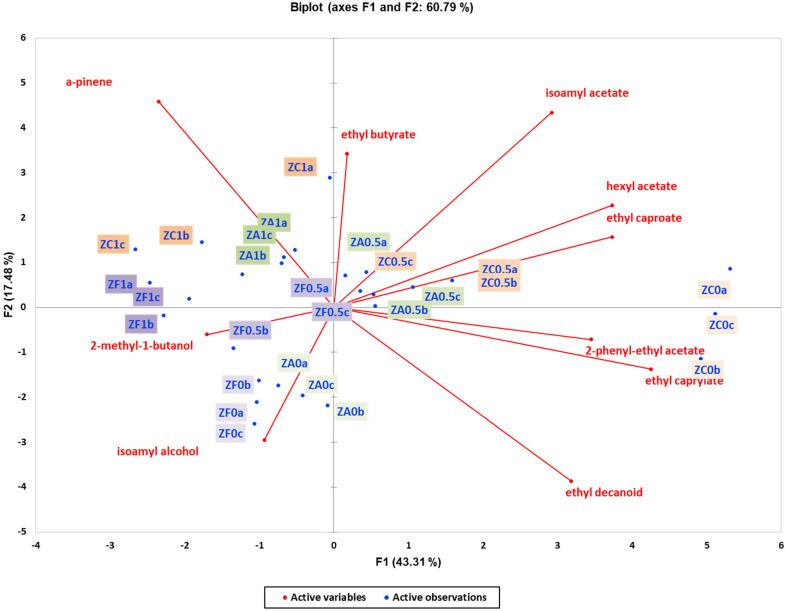
Unsupervised classification using principal component analysis on metabolomic data (volatile compounds) from the experimental wines produced by the ZymafloreX5 yeast. Letters of the samples in the Biplot indicate the ID: Z—ZymafloreX5, F—French oak, A—American oak, C—control (no oak), 0—no resin, 0.5—0.5 g/L resin, 1—1 g/L resin, a; b; c—replication. Samples in the score plots were coloured according to the resin treatments (0, 0.5, and 1 g/L) and oak treatment (no oak—orange, French oak—purple, American oak—green). Sequential increase in the resin concentration is presented with darker colours (0 g/L resin—pale, 0.5 g/L— medium dark, and 1 g/L—dark).

**Figure 6:**
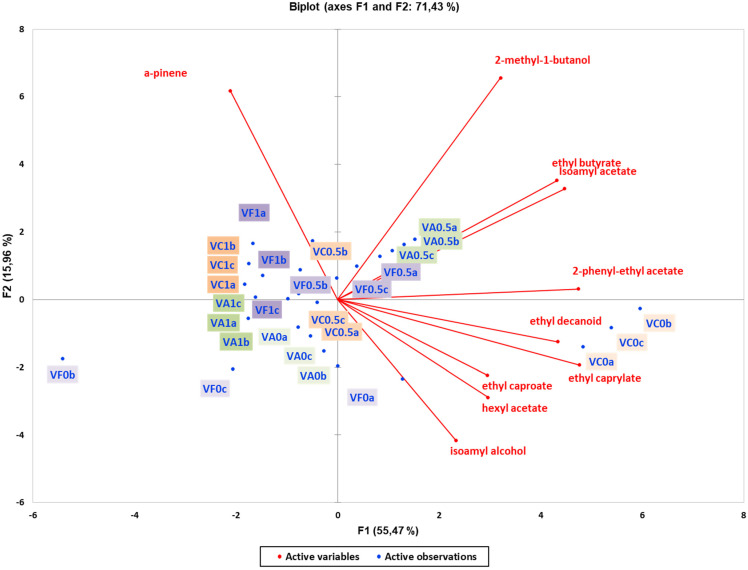
Unsupervised classification using principal component analysis on metabolomic data (volatile compounds) from the experimental wines produced by **the** Vivace yeast. Letters of the samples in the Biplot indicate the ID: V—Vivace, F—French oak, A—American oak, C—control (no oak), 0—no resin, 0.5—0.5 g/L resin, 1—1 g/L resin, a; b; c—replication. Samples in the score plots were coloured according to the resin treatments (0, 0.5, and 1 g/L) and oak treatment (no oak—orangek, French oak—purple, American oak—green). Sequential increase in the resin concentration is presented with darker colours (0 g/L resin—pale, 0.5 g/L medium—dark, and 1 g/L—dark).

The PCA biplot model for wines based on volatile compounds was constructed for two yeast strains (Figure 5 and Figure 6). The PCA score plot for wines produced with Vivace yeast explained 60.79% of the variance and indicated that the resin effect was the main factor of discrimination, with a separation along the PC1 axis for volatile compounds (Figure 5). Similarly, the PCA score plot for wines produced with Zymaflore X5 yeast explained 71.43% of the variance, with separation along PC1 also highlighting the impact of resin concentration on volatile compounds (Figure 6). 

The projection of α-pinene showed that this specific volatile compound was responsible for distinguishing between non-resinated and resinated wine samples, with a decrease in α-pinene observed across all wines with varying resin concentrations (Figure 5). In the Zymaflore X5 and Vivace yeast experiment, the biplot further demonstrated that wines were clearly clustered according to resin concentration with α-pinene levels decreasing as resin concentration decreased (Figure 6). In both PCA (Figure 5 and Figure 6), wine samples with higher concentrations of resin (1 g/L) are grouped in the second quadrant. Furthermore, it is evident from both PCA plots that in each yeast strain, wines without resin are characterised by specific volatile compounds such as ethyl decanoate, 2-phenyl-ethyl acetate, and ethyl caprylate (Figure 5 and Figure 6).

### 3.3. Univariate Statistics

#### 3.3.1. Colour and Phenolic Parameters, Photometrics

The oenological parameters such as Total Phenolic Index, wine colour intensity, oxidation rate (k factor), and antioxidant activity using the DPPH assay revealed differences among the experimental wines produced by the Vivace and Zymaflore X5 yeasts (Figures S1 and S2). Overall, the resin and oak chips did not consistently affect the colour and phenolic parameters of the wines produced by both yeast strains (Figures S1 and S2). However, wines produced with Vivace yeast showed the highest colour intensity when resin was added. According to the data in Figures S1 and S2, the interaction effect between resin and oak is statistically significant for all parameters, including Total Phenolic Index, Colour Intensity, k factor, and DPPH.

#### 3.3.2. Organic Acids and Phenolic Compounds

The HPLC analysis of the samples allowed the detection of 11 phenolic compounds and four organic acids. A significant diversification in terms of phenolic compounds and organic acids was observed, and a two-way ANOVA was carried out for each volatile compound of the volatile dataset, using resin and oak chips as factors in the two yeast strains. The interaction between the two factors was also considered. The *p*-values of the two-way ANOVA null hypothesis are reported for both the factors and their interaction. A *p*-value of 0.05 was considered as the significance threshold. Overall, it can be deduced that the utilisation of resin and oak chips as treatment agents for wines has resulted in an increase in the observed organic acids and phenolic compounds in both yeast strains.

Based on the data reported in Figures S3 and S4, regarding the organic acids, the interaction effect between the resin and the oak chips is statistically significant for 3 molecules (tartaric, malic, and acetic acid), while the molecule not affected by this interaction is the succinic acid in wines produced in both yeast strains. However, resin concentration plays a significant role in determining the levels of succinic acid in wines (Figures S3 and S4).

Each of the 11 wine phenolic components in each of the wine types varied considerably in concentration. The compounds for which significant oak-origin effects were observed are given in Figures S5–S8. There was a general trend across all the wines towards a higher concentration of syringaldehyde, ellagic acid, and vanillic acid in those produced with the addition of American and French oak chips (Figures S5 and S7). In contrast, the wines that were not treated with oak chips exhibited higher levels of syringic and *p*-coumaric acid (Figures S6 and S8). Statistically significant oak and resin effects were observed for the above-targeted five compounds in wines, and the interaction of the two factors’ effects was also significant (Figures S5–S8).

#### 3.3.3. Volatile Compounds

This study also aimed to evaluate the influence of resin and oak chips on the volatile compounds in experimental wines. The data confirmed that the interaction between resin and oak chips can generate varying levels of volatile compounds. Significant diversification in the amount of volatile compounds was observed, and a two-way ANOVA was performed for each volatile molecule in the dataset, using resin and oak chips as factors. The interaction between these two factors was also considered. The levels of extracted volatile compounds varied quantitatively depending on the resin dose and the type of oak chip treatment, with significant decreases in esters being particularly notable.

Based on the data reported in Figures S9–S11, the interaction effect between the resin and the oak chips is statistically significant for four molecules (ethyl decanoate, 2 methyl-1-butanol, 2-phenyl-ethyl acetate, and ethyl butyrate) in wines produced by Vivace yeast and for four compounds (2 methyl-1-butanol, isoamyl acetate, ethyl caproate, and hexyl acetate) in wines produced by Zymaflore X5 yeast. Despite the fact that, in wines produced by Vivace yeast, no significant interaction of resin and oak chips was recorded (except for a-pinene, for wines produced by Zymaflore X5, four volatile compounds were significantly influenced by oak chips (a-pinene, ethyl caproate, hexyl acetate, and 2-phenyl ethyl acetate) as well as five by resin (ethyl decanoate, a-pinene, isoamyl acetate, hexyl acetate, and ethyl caprylate), respectively (Figures S9–S11). Resin significantly influences the amount of α-pinene, increasing its concentration in wines treated with a higher dose of resin (Figure S9a,b). Furthermore, the results confirmed that the concentration of esters (including ethyl decanoate, ethyl caprylate, ethyl caproate, hexyl acetate, and ethyl butyrate) decreased with increasing resin doses in wines produced by both yeast strains. For example, all esters were detected in higher concentrations in wines without oak and resin compared to those treated with resin and oak.

#### 3.3.4. Sensory Analysis

A two-way ANOVA (resin, oak chips) with interaction and a multiple comparison of means by Tukey’s test was performed. The sensory analysis revealed that 8 descriptors from Vivace yeast and 11 descriptors from Zymaflore X5 yeast showed significant differences according to the two-way ANOVA. The interaction between the two factors was also considered. Table S1 reports the *p*-values of the two-way ANOVA for both factors and their interaction, with a significance threshold set at 0.05 (*p* > 0.05 indicated as n.s.). The panellists scored the sensory descriptors on a structured scale from 0 (no perception) to 5 (highest perception).

**Figure 7:**
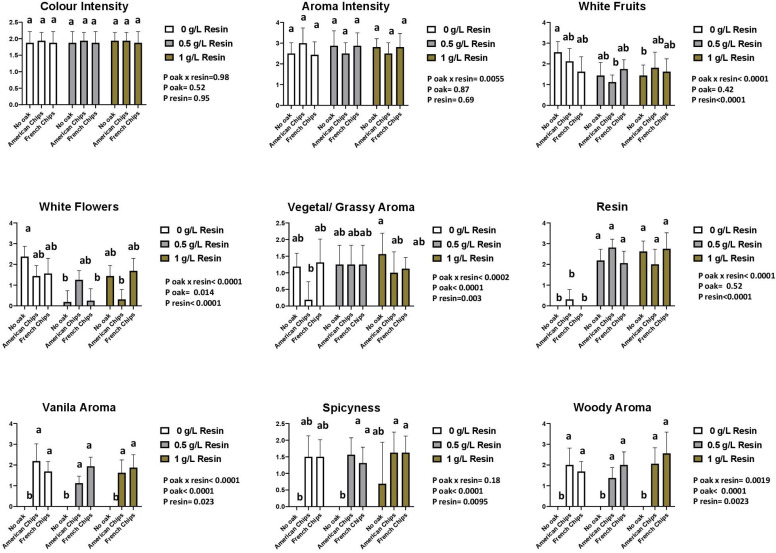
Histogram of visual and odour descriptors regarding wines produced by Vivace yeast. Letters above the bars indicate significant differences (*p* < 0.05) between each wine as determined by Tukey’s post hoc analysis.

**Figure 8:**
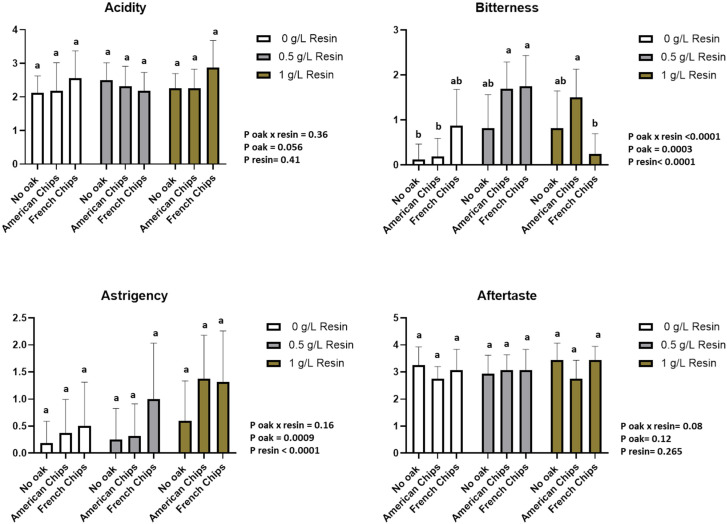
Histogram of taste descriptors regarding wines produced by Vivace yeast. Letters above the bars indicate significant differences (*p* < 0.05) between each wine as determined by Tukey’s post hoc analysis.

**Figure 9:**
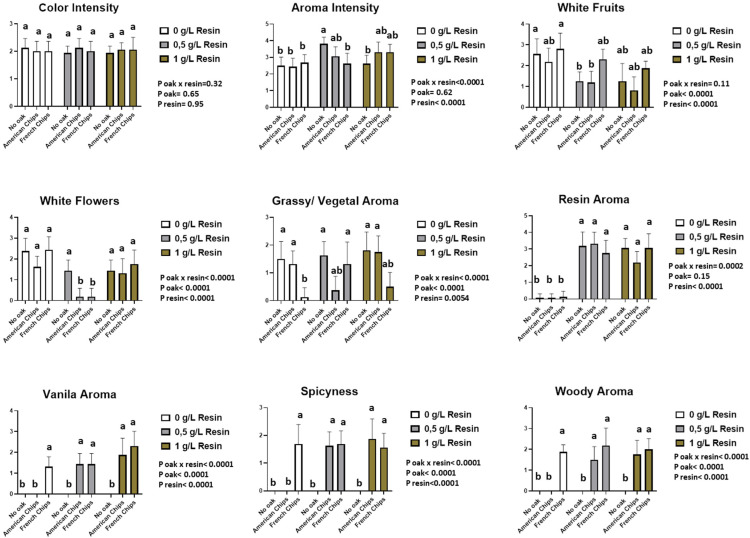
Histogram of visual and odour descriptors regarding wines produced by Zymaflore X5 yeast. Letters above the bars indicate significant differences (*p* < 0.05) between each wine as determined by Tukey’s post hoc analysis.

**Figure 10:**
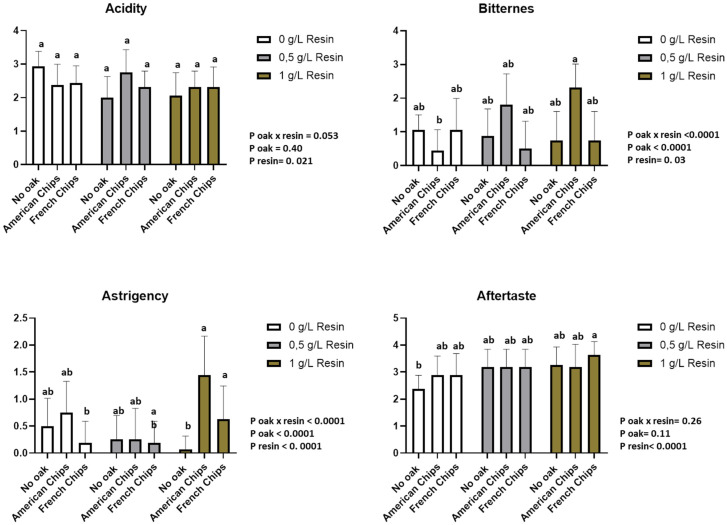
Histogram of taste descriptors regarding wines produced by Zymaflore X5 yeast. Letters above the bars indicate significant differences (*p* < 0.05) between each wine as determined by Tukey’s post hoc analysis.

Table S1 presents the analysis of variance based on the data provided by the panellists. Most odour descriptors are significantly affected by resin and oak chips, as well as by their interaction. However, no significant differences were observed among the wines in terms of colour intensity, acidity, and aftertaste descriptors. Additionally, the sensory analysis data are presented in Figure 7, Figure 8, Figure 9 and Figure 10.

The profile assessment of the aroma showed significant differences between the two factors (resin and oak chips) and the interaction of the factors among the tested wine samples. The treatment with resin and oak chips as well as the interaction of these two factors revealed an increase in spiciness and vanilla and woody aroma (Figure 7 and Figure 8). Wines produced with Zymaflore X5 yeast and treated with French oak chips exhibited higher levels of woody and vanilla aromas compared to those treated with American oak chips when no resin was added. Regarding spiciness, Zymaflore X5 yeast reduced the spiciness in wines without resin and treated with American oak chips (Figure 9), compared to wines produced with Vivace yeast (Figure 7). For the resin aroma assessment, wines produced by both yeast strains had higher scores for resinated samples, while non-resin-treated samples received lower scores (Figure 7 and Figure 9). However, statistical analysis showed no significant differences among different resin doses. Concerning taste descriptors such as acidity and aftertaste, no differences were observed. In wines treated with American oak chips using both yeasts, bitterness levels were higher compared to those treated with French oak chips when a higher dose of resin was used (Figure 8 and Figure 10). The astringency of wines produced with Vivace yeast increased with higher resin doses, with French oak-treated wines showing higher levels of astringency, although no significant differences were recorded (Figure 8). Conversely, wines produced with Zymaflore X5 yeast recorded higher astringency levels when treated with American oak chips. Additionally, wines treated with 0.5 g/L resin had the lowest astringency levels, even lower than wines without any resin (Figure 10).

Overall, the wine samples without resin were characterised by a more fruity and floral odour and taste profile. In contrast, the resinated wines exhibited a more complex profile, with noticeable bitterness and resin aroma (Figure 7, Figure 8, Figure 9 and Figure 10).

## 4. Discussion

The impact of resin concentration, yeast selection, and the type of oak chips on the quality of “Retsina”, the traditional Greek resinated wine, was investigated. The outcomes of this study provide valuable insights into the way these three factors affect the chemical composition and sensory properties of Savatiano resinated wines.

**Effect of the Factors on the Winemaking Process**

An initial analysis of the classical oenological data (Table 2) revealed that the alcohol content across all experimental wines remained relatively consistent, with no statistically significant differences observed. The uniformity observed can be partially attributed to the simultaneous harvesting of the grapes for microvinifications, which ensured consistent ripeness and quality throughout the batch. This also suggests a high degree of standardisation in the winemaking process across all treatments. The residual sugar levels (Table 2) demonstrate that the fermentation process was successfully completed in all treatments, regardless of resin concentration or oak chip usage. This finding indicates that neither the addition of resin nor the use of oak chips impeded the fermentation process.

**Impact of yeast strain on Chemical Composition and Sensory Profile**

A critical finding of this research is the significant role that yeast strain plays in the chemical composition of “Retsina”. Variations in phenolic compounds, organic acids, and volatile compounds were observed depending on whether the Vivace or Zymaflore X5 yeast strain was used. This yeast-induced differentiation was visualised through Principal Component Analysis (PCA), where PC1 and PC2 effectively grouped wine samples according to their yeast strain (Figure 1 and Figure 2).

Notably, wines fermented with the Zymaflore X5 yeast were characterised by higher concentrations of organic acids such as tartaric, malic, acetic, and succinic acids, which were positively correlated with this yeast strain.

The results concerning the concentrations of organic acids can provide a rationale for the outcomes regarding the total and volatile acidity of the experimental wines, as illustrated in Table 2. Reviewing Table 2, it becomes apparent that regardless of the presence of resin or oak, there was a statistically significant increase in total acidity, ranging from 0.2 to 0.5 g/L tartaric acid, and in volatile acidity, ranging from 0.10 to 0.20 g/L acetic acid, observed in wines fermented with the Zymaflore X5 strain compared to those produced with the Vivace strain. These findings demonstrate the positive impact of the Zymaflore X5 strain on acidity, which plays a crucial role in preserving sensory freshness and ensuring proper ageing of wines, especially for the Savatiano variety, known for its low acidity [33,34,35,36].

Regarding volatile acidity, it is noteworthy that all wines produced by the two commercial yeast strains exhibited no undesirable levels of acetic acid, which remained well below 0.6 g/L.

In terms of volatiles, the Zymaflore X5 yeast strain produced higher concentrations of aromatic compounds such as ethyl caprylate, 2-phenyl ethyl acetate, ethyl caproate, and ethyl decanoate in wines without resin and oak additions (Figure 2). These compounds contribute to fruity and floral aromas, thereby enhancing the aromatic profile of the wine. In contrast, Vivace yeast showed elevated levels of hexyl acetate and isoamyl alcohol, particularly when oak and resin were introduced (Figure 2).

The yeast strain had a significant impact on the sensory profile of the wines.

Vivace yeast contributed to higher levels of woody and vanilla aromas when the wines were treated with American chips with no resin addition. Also, wines produced with Zymaflore X5 yeast exhibited lower spiciness compared to those produced with Vivace yeast when treated with American oak chips without resin, indicating that Zymaflore X5 yeast moderates this attribute (Figure 7 and Figure 9).

**Impact of Resin on Chemical Composition and Sensory Profile**

The addition of resin had a profound effect on the chemical composition of the wines, influencing both phenolic compounds and volatile profiles. Resin, primarily known for contributing to the characteristic “Retsina” aroma, significantly increased the levels of α-pinene, a volatile compound derived from resin. This compound was found in higher concentrations in wines with a higher concentration of resin, contributing to the distinctive resinous aroma profile of the wines (Figure S9).

In terms of phenolic composition, the interaction between resin and oak chips significantly affected the concentrations of several key phenolic compounds. Wines treated with resin but without oak exhibited higher levels of phenolic compounds such as *p*-coumaric acid, syringic acid, and (-)-epicatechin (Figures S6 and S8), which are typically associated with antioxidant properties and contribute to the structural complexity of the wine. Resin also had a notable impact on the concentration of succinic acid, which was significantly increased in wines produced by Zymaflore X5 yeast, regardless of the presence or absence of oak chips (Figure S4). 

Moreover, there was a noticeable impact of resin on ester formation, with a suppression of certain fruity esters like isoamyl acetate, hexyl acetate, and ethyl caprylate in oak-treated wines as resin concentration increased (Figures S10 and S11). This suggests that resin may inhibit the formation of esters, leading to a reduction in the fruity character of the wine. This outcome aligns with the sensory analysis results (Figures S7 and S9), where wine samples without resin were described as having a more pronounced fruity and floral odour and taste profile, supporting the connection between ester concentrations and the aromatic profile of the wines. As expected, wines treated with resin had significantly higher scores for resin aroma compared to non-resin-treated samples (Figures S7 and S9). However, statistical analysis revealed that different resin doses (0.5 g/L and 1 g/L) did not result in significant differences in resin aroma perception.

Wines treated with resin generally exhibited increased bitterness and a more complex profile, especially when combined with oak chips (Figures S8 and S10). However, wines treated with the lower resin dose (0.5 g/L) showed lower astringency levels and, in the case of wines from Zymaflore X5 yeast, lower than even the non-resinated samples, suggesting that resin can modulate mouthfeel without overwhelming the wine (Figure S10). In contrast, higher resin doses led to increased astringency, especially in wines treated with oak (Figures S8 and S10). 

**Impact of Oak on Chemical Composition and Sensory Profile**

This study provides a comprehensive analysis of the impact of oak addition on the chemical composition of Retsina. Previous research has established that oak chips significantly influence the phenolic composition of white dry wines. For instance, Li et al. [37] demonstrated that French oak chips enhance the total phenolic and flavonoid content, thereby increasing the antioxidant capacity of the wine. This is corroborated by Galdo et al. [38] who reported that both oak and cherry wood chips boost the phenolic content and colour intensity of white wines. Furthermore, Laqui-Estaña et al. [39] highlighted the influence of the oak wood format, grape variety, and ageing time on the phenolic enrichment of wines. Collectively, these studies underscore the significant role of oak chips in affecting the quality and complexity of white dry wines.

In this study, distinct phenolic profiles were observed in wines produced with oak chips compared to those without oak. Specifically, significant increases were observed in vanillic acid, ellagic acid, syringaldehyde, and coniferaldehyde contents (Figure S7). Conversely, wines without oak chips exhibited higher levels of syringic acid and *p*-coumaric acid (Figure S6), suggesting that the absence of oak allows for the preservation or even enhancement of these phenolic compounds. These findings are consistent with existing literature, which highlights the role of oak chips in introducing specific phenolic compounds into wine, thereby altering its chemical composition and potentially its sensory attributes [25,37,38,39,40].

Regarding the volatile compounds of the wines produced from both yeast strains, the addition of oak chips resulted in lower levels of certain fermentation aroma compounds such as isoamyl acetate, hexyl acetate, ethyl caprylate, and 2-phenyl-ethyl acetate when no resin was added (Figures S10 and S11). These findings suggest a potential retention of fruity characteristics in these wines, although there were no statistically significant differences among sensory data. This indicates that while the chemical analysis shows clear differences in volatile profiles, these differences were not perceptible by the sensory panel, indicating the complex relationship between chemical composition and sensory perception in wine—a phenomenon that has been observed in other studies as well and may be attributed to interactions between the volatile compounds in wine matrix compounds [41,42].

However, the results are contrary to the findings of Palomo et al. [43], who observed that wines fermented with oak chips generally contained higher concentrations of the ethyl esters of fatty acids (ethyl butanoate, ethyl hexanoate, ethyl octanoate, and ethyl decanoate) and ethyl, hexyl, and isoamyl acetates in comparison to the control wine. Other studies also report an increase in the fruity character of wines fermented with oak chips [44,45]. In these studies, the authors suggested that oak chips act as a carrier for the yeast cells, which gives rise to an effect similar to that of immobilised cells. Despite the increase in esters observed in their studies, they reported less fruity aromas in the sensory assessment of the wines fermented in the presence of oak woods.

The contradictory results obtained in this study may be attributed to the interaction between the yeast strain and the oak chips. Further research is needed to fully understand these interactions and their impact on wine aroma and flavour.

The type of oak significantly influenced the phenolic composition of wines. In every case, wines produced with American oak chips had higher concentrations of ellagic acid compared to those made with French oak chips (Figures S5 and S7).

The impact of oak chips on the sensory characteristics of the wines was notable in our study, particularly in terms of enhancing woody and vanilla aromas and spiciness, as depicted in Figures S7 and S9. These findings are consistent with the literature [46,47]. 

More specifically, French oak chips were found to significantly affect the vanilla, spicy, and woody aromas in wines produced with Zymaflore X5 yeast, particularly in the absence of resin (Figure S9). This suggests that the choice of oak type can have a discernible impact on the aromatic characteristics of the resulting wine. The presence or absence of resin appears to modulate this effect, although further research is needed to fully understand the interaction between resin addition and oak type in shaping wine aroma.

The differences in aroma descriptors can be attributed to variations in the content of specific compounds present in oak wood, such as trans and cis-β-methyl-γ-octalactone, known for their contribution to the “woody” character in wines aged with oak woods, as suggested by previous research [48,49], and other wood-related compounds described in other studies [25,50,51].

Wines treated with American oak chips tended to show a spicier aroma. However, in wines fermented with Zymaflore X5, the spiciness was reduced when no resin was added. In contrast, wines produced with Vivace yeast showed a higher spiciness when treated with American oak (Figures S7 and S9).

Wines treated with American oak chips showed higher levels of astringency, particularly in wines produced with Zymaflore X5 yeast. On the other hand, French oak increased astringency in Vivace yeast wines, especially at higher resin doses, though these increases were not statistically significant (Figure S10).

## 5. Conclusions

This study provides significant insights into the impact of resin concentration, yeast selection, and oak chip type on the chemical composition and quality of Retsina, the traditional Greek resinated wine. Yeast strain notably influenced Retsina’s chemical profile, with Zymaflore X5 fermentations showing higher organic acid concentrations, thereby enhancing acidity and sensory freshness. The addition of oak significantly altered the phenolic profiles, with American oak increasing ellagic acid concentrations, while Retsina without oak exhibited higher levels of syringic and *p*-coumaric acids. Sensory analysis indicated that oak chips enhanced woody, vanilla, and spicy aromas in Retsina. Additionally, the inclusion of resin increased α-pinene levels, contributing to the distinctive pine aroma of Retsina, while reducing ester concentrations, which are typically associated with fruity aromas. The interaction between yeast and oak was complex, affecting the phenolic and volatile profiles in ways that did not always align with sensory perceptions. The findings underscore the importance of strategic yeast and oak selection to optimise Retsina quality. Future research should further explore these interactions and innovative approaches to maintain Retsina quality amidst evolving climatic conditions, contributing to the broader understanding of optimising this traditional Greek wine for global markets.

## Figures and Tables

**Table 1:** Physicochemical parameters of Savatiano Grape Must.

Savatiano Must	
Weight/Berry (g)	2.45
TSS (°Brix)	22.2
Density	1.095
Total Acidity (g/L Tartaric Acid)	4.43
pH	3.22
YAN (mg/L)	150

**Table 2:** Oenological Parameters of Experimental Wines Produced by Vivace and Zymaflore X5 Yeasts with Different Oak Chip Treatments (French and American) and Resin Concentrations (0.5 g/L and 1 g/L). Data represent mean and standard errors of the means. P_oak_: probability value for the addition of oak chips (American and French); P_resin_: probability value for the resin (0.5 and 1 g/L) addition; P_oak×resin_: probability value for the oak × resin interaction. *p* values > 0.05 indicate lack of significant effect. Different letters between columns indicate significant differences at *p* < 0.05 (Tuckey multiple range test).

Vivace										
	0 g/L Resin			0.5 g/L Resin			1 g/L Resin			
	No Chips	American	French	No Chips	American	French	No Chips	American	French	*p* Values
Alcoholic Volume (*v/v* %)	12.8 ± 0.10 a	12.8 ± 0.10 a	12.9 ± 0.10 a	12.9 ± 0.10 a	12.9 ± 0.10 a	12.9 ± 0.10 a	12.9 ± 0.10 a	12.9 ± 0.10 a	12.8 ± 0.1 a	*p* _oak×resin_ = 0.13 *p* _oak_ = 0.53 *p* _resin_ = 0.13
pH	3.42 ± 0.01 a	3.37 ± 0.01 bc	3.38 ± 0.00 b	3.36 ± 0.00 c	3.37 ± 0.00 bc	3.38 ± 0.01 b	3.37 ± 0.01 bc	3.38 ± 0.00 b	3.37 ± 0.0 bc	*p* _oak×resin_ = 0.001 *p* _oak_ = 0.094 *p* _resin_ = 0.0018
Total Acidity (g/L of Tartaric Acid)	5.7 ± 0.10 a	5.7 ± 0.00 a	5.7 ± 0.10 a	5.8 ± 0.00 a	5.7 ± 0.00 a	5.8 ± 0.00 a	5.8 ± 0.00 a	5.8 ± 0.00 a	5.8 ± 0.00 a	*p* _chips×resin_ = 0.55 *p* _oak_ = 0.29 *p* _resin_ = 0.06
Volatile Acidity (g/L Acetic Acid)	0.35 ± 0.01 a	0.38 ± 0.10 ab	0.36 ± 0.20 a	0.31 ± 0.20 a	0.40 ± 0.07 a	0.34 ± 0.08 a	0.34 ± 0.03 a	0.33 ± 0.03 a	0.32 ± 0.06 a	*p* _oak×resin_ = 0.47 *p* _oak_ = 0.33 *p* _resin_ = 0.36
Residual Sugars (g/L)	1.09 ± 0.03 a	1.09 ± 0.03 a	1.08 ± 0.02 a	1.11 ± 0.03 a	1.16 ± 0.07 a	1.19 ± 0.07 a	1.11 ± 0.03 a	1.03 ± 0.08 a	1.15 ± 0.06 a	*p* _oak×resin_ = 0.23 *p* _oak_ = 0.19 *p* _resin_ = 0.11
Free SO_2_ (mg/L)	19.66 ± 0.57 a	18.33 ± 1.52 a	21.00 ± 1.00 a	20.00 ± 1.00 a	20.00 ± 1.00 a	21.33 ± 0.57 a	20.00 ± 1.00 a	20.66 ± 0.57 a	21.66 ± 0.57 a	*p* _oak×resin_ = 0.22 *p* _oak_ = 0.20 *p* _resin_ = 0.11
**Zymaflore**										
	**0 g/L Resin**			**0.5 g/L Resin**			**1 g/L Resin**			
	**No Chips**	**American**	**French**	**No Chips**	**American**	**French**	**No Chips**	**American**	**French**	***p*** **Values**
Alcoholic Volume (*v/v*%)	12.90 ± 0.20 a	12.70 ± 0.10 a	12.80 ± 0.14 ab	12.70 ± 0.14 bc	12.70 ± 0.20 a	12.80 ± 0.0 a	12.60 ± 0.20 a	12.75 ± 0.20 a	12.75 ± 0.07 a	*p* _oak×resin_ = 0.90 *p* _oak_ = 0.74 *p* _resin_ = 0.74
pH	3.39 ± 0.01 a	3.35 ± 0.01 c	3.36 ± 0.01 bc	3.38 ± 0.00 ab	3.36 ± 0.01 bc	3.36 ± 0.00 c	3.35 ± 0.01 c	3.36 ± 0.00 c	3.36 ± 0.01 bc	*p* _oak×resin_ = 0.016 *p* _oak_ = 0.012 *p* _resin_ = 0.077
Total Acidity (g/L of Tartaric Acid)	6.1 ± 0.10 ab	6.1 ± 0.00 b	6.1 ± 0.00 b	6.2 ± 0.10 ab	6.1 ± 0.00 b	6.0 ± 0.20 b	6.3 ± 0.00 a	6.1 ± 0.00 b	6.1 ± 0.00 b	*p* _oak×resin_ = 0.45 *p* _oak_ = 0.05 *p* _resin_ = 0.47
Volatile Acidity (g/L Acetic Acid)	0.49 ± 0.01 a	0.50 ± 0.01 a	0.45 ± 0.03 a	0.51 ± 0.04 a	0.49 ± 0.01 a	0.49 ± 0.00 a	0.49 ± 0.01 a	0.51 ± 0.06 a	0.50 ± 0.00 a	*p* _oak×resin_ = 0.81 *p* _oak_ = 0.77 *p* _resin_ = 0.91
Residual Sugars (g/L)	1.12 ± 0.01 c	1.82 ± 0.07	1.46 ± 0.63 abc	1.70 ± 0.41 ab	1.66 ± 0.00 abc	1.26 ± 0.01 bc	2.04 ± 0.02 aab	1.55 ± 0.10 abc	1.50 ± 0.01 abc	*p* _oak×resin_ = 0.837 *p* _oak_ = 0.15 *p* _resin_ = 0.17
Free SO_2_ (mg/L)	20.33 ± 1.53 a	19.00 ± 2.64 a	20.66 ± 1.15 a	20.00 ± 1.00 a	20.00 ± 1.00 a	19.66 ± 0.55 a	20.50 ± 0.50 a	20.00 ± 1.00 a	21.50 ± 0.50 a	*p* _oak×resin_ = 0.137 *p* _oak_ = 0.14 *p* _resin_ = 0.15

## Data Availability

The data presented in this study are available on request from the corresponding authors due to privacy and ethical considerations.

## Supplementary Materials

The following supporting information can be downloaded at: https://www.mdpi.com/article/10.3390/foods13213376/s1, Figure S1: Plots presenting Color and phenolic parameters of the experimental wines in response to two-way ANOVA, different resin treatments (0 g/L, 0.5 g/L and 1 g/L of resin) and different oak chips(no Chips, American oak and French oak), produced with the Vivace yeast; Figure S2: Plots presenting Colour and phenolic parameters of the wines in response to two-way ANOVA, different resin treatments (0 g/L, 0.5 g/L and 1 g/L of resin) and different oak chips(no Chips, American oak and French oak), produced with the Zymaflore X5 yeast; Figure S3: Plots presenting Organic acids of the experimental wines in response to two-way ANOVA, different resin treatments (0 g/L, 0.5 g/L and 1 g/L of resin) and different oak chips (no Chips, American oak and French oak), produced with the Vivace yeast; Figure S4: Plots presenting Organic acids of the experimental wines in response to two-way ANOVA, different resin treatments (0 g/L, 0.5 g/L and 1 g/L of resin) and different oak chips (no Chips, American oak and French oak), produced with the Zymaflore X5 yeast; Figure S5: Plots presenting Phenolic compounds of the experimental wines in response to two-way ANOVA, different resin treatments (0 g/L, 0.5 g/L and 1 g/L of resin) and different oak chips (no Chips, American oak and French oak), produced with the Vivace yeast; Figure S6: Plots presenting Phenolic compounds of the experimental wines in response to two-way ANOVA, different resin treatments (0 g/L, 0.5 g/L and 1 g/L of resin) and different oak chips (no Chips, American oak and French oak), produced with the Vivace yeast; Figure S7. Plots presenting Phenolic compounds of the experimental wines in response to two-way ANOVA, different resin treatments (0 g/L, 0.5 g/L and 1 g/L of resin) and different oak chips (no Chips, American oak and French oak), produced with the Zymaflore X5 yeast; Figure S8: Plots presenting Phenolic compounds of the experimental wines in response to two-way ANOVA, different resin treatments (0 g/L, 0.5 g/L and 1 g/L of resin) and different oak chips (no Chips, American oak and French oak), produced with the ZYMAFLORE X5 yeast; Figure S9: Plots presenting volatile compounds of the experimental wines Trend plots presenting Phenolic compounds of the experimental wines in response to two way ANOVA, different resin treatments (0 g/L, 0.5 g/L and 1 g/L of resin) and different oak chips (no Chips, American oak and French oak), produced with the Vivace (a) and Zymaflore X5 (b) yeast; Figure S10: Plots presenting Volatile compounds of the experimental wines in response to two-way ANOVA, different resin treatments (0 g/L, 0.5 g/L and 1 g/L of resin) and different oak chips (no Chips, American oak and French oak), produced with the Vivace yeast; Figure S11: Plots presenting Volatile compounds of the experimental wines in response to two-way ANOVA, different resin treatments (0 g/L, 0.5 g/L and 1 g/L of resin) and different oak chips (no Chips, American oak and French oak), produced with the ZYMAFLORE X5 yeast; Figure S12: Experimental design for assessing the impact of resin concentration, oak chip type, and yeast strain on Retsina wine; Table S1: Multiple analysis of variance to analyze the influence of the resin treatment and the oak chips level and their interaction in the aromatic series.
