# Supplementary material for: Optimisation of Retsina Wine Quality: Effects of Resin Concentration, Yeast Strain, and Oak Chip Type
Source: Foods. 2024 Oct 24;13(21):3376. doi: 10.3390/foods13213376 (PMC11545637; doi:10.3390/foods13213376)
Supplement: Supplementary file 1 [file foods-13-03376-s001.zip › foods-3237150-supplementary.pdf]

Supplementary

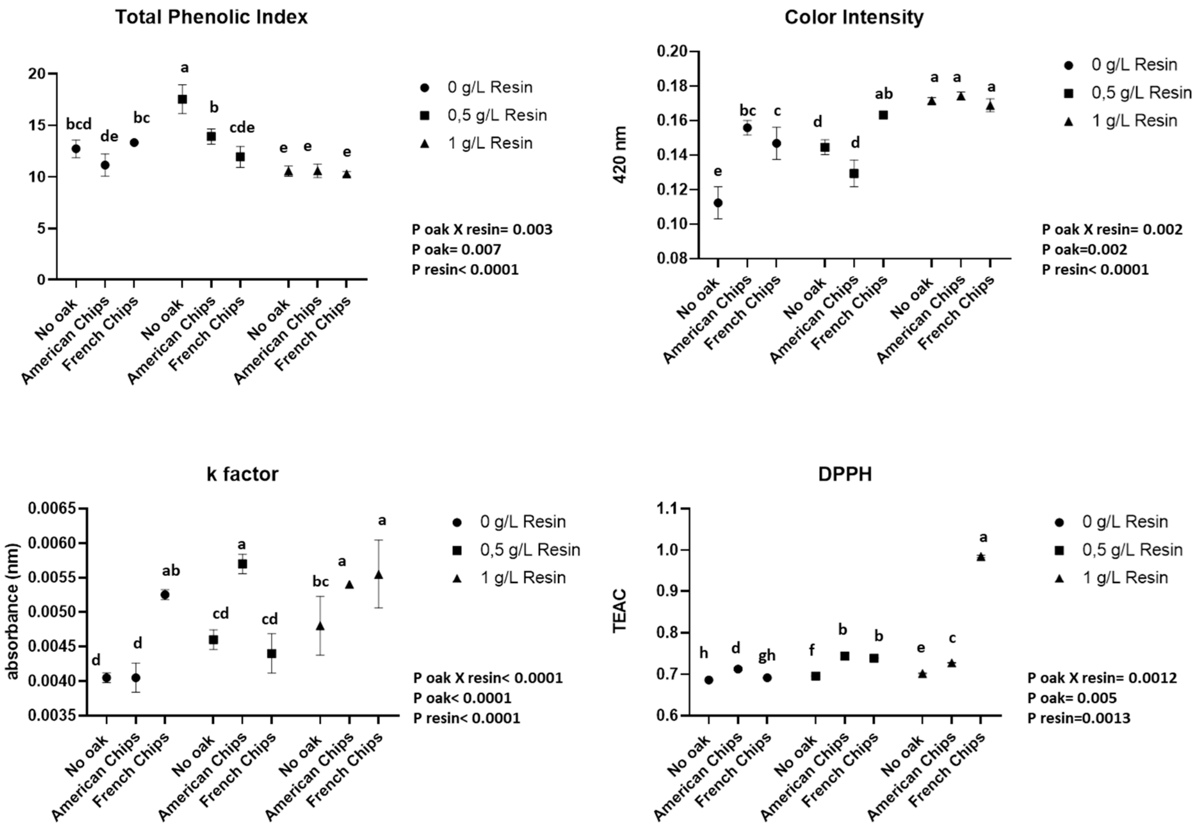

Figure S1. Plots presenting Color and phenolic parameters of the experimental wines in response to two-way ANOVA, different resin treatments (0 g/L, 0.5 g/L and 1 g/L of resin) and different oak chips(no Chips, American oak and French oak), produced with the Vivace yeast. Data represent mean and standard deviations represented by error bars. P_{oak}, probability value for the addition of oak chips (American and French); P resin, probability value for the resin (0.5 and 1 g/L) addition; P oak x resin, probability value for the oak × resin interaction. P values higher than 0.05 indicate lack of significant effect. Different letters between columns indicate significant differences at $p < 0.05$. (Tuckey multiple range test).

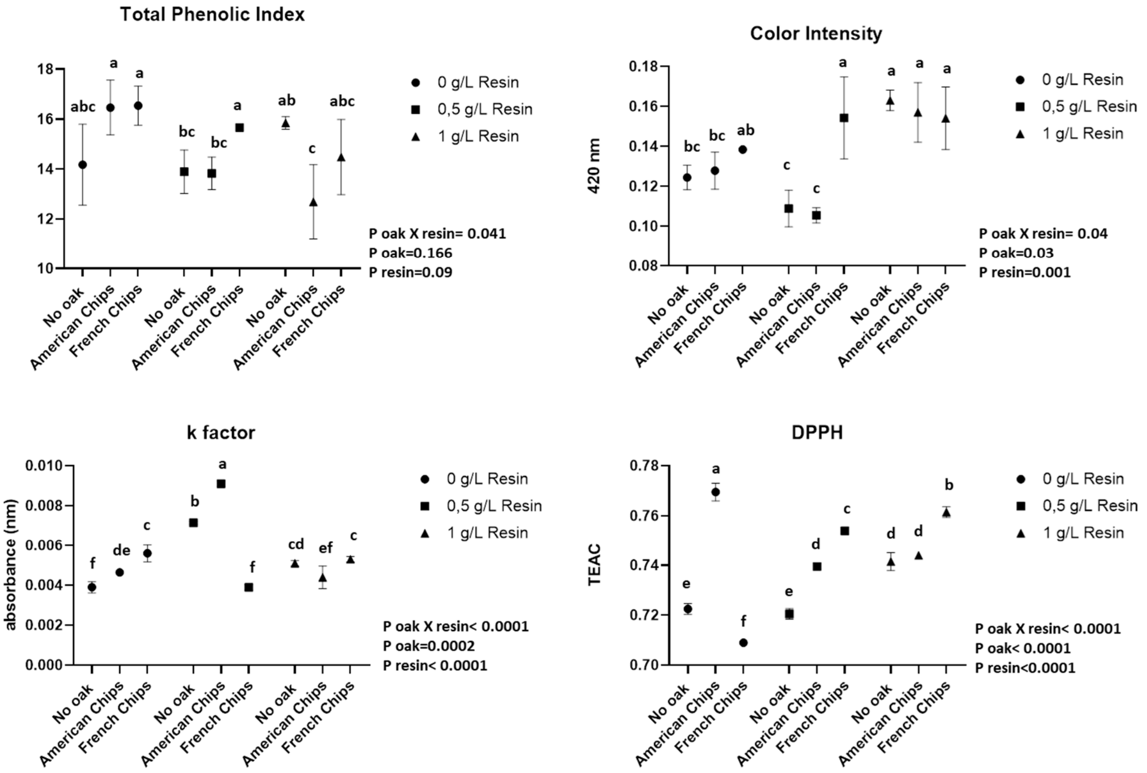

Figure S2. Plots presenting Colour and phenolic parameters of the wines in response to two-way ANOVA, different resin treatments (0 g/L, 0.5 g/L and 1 g/L of resin) and different oak chips(no Chips, American oak and French oak), produced with the Zymaflore X5 yeast. Data represent mean and standard deviations represented by error bars. Poak, probability value for the addition of oak chips (American and French); P resin, probability value for the resin (0.5 and 1 g/L) addition; P oak x resin, probability value for the oak × resin interaction. P values higher than 0.05 indicate lack of significant effect. Different letters between columns indicate significant differences at $p < 0.05$. (Tuckey multiple range test).

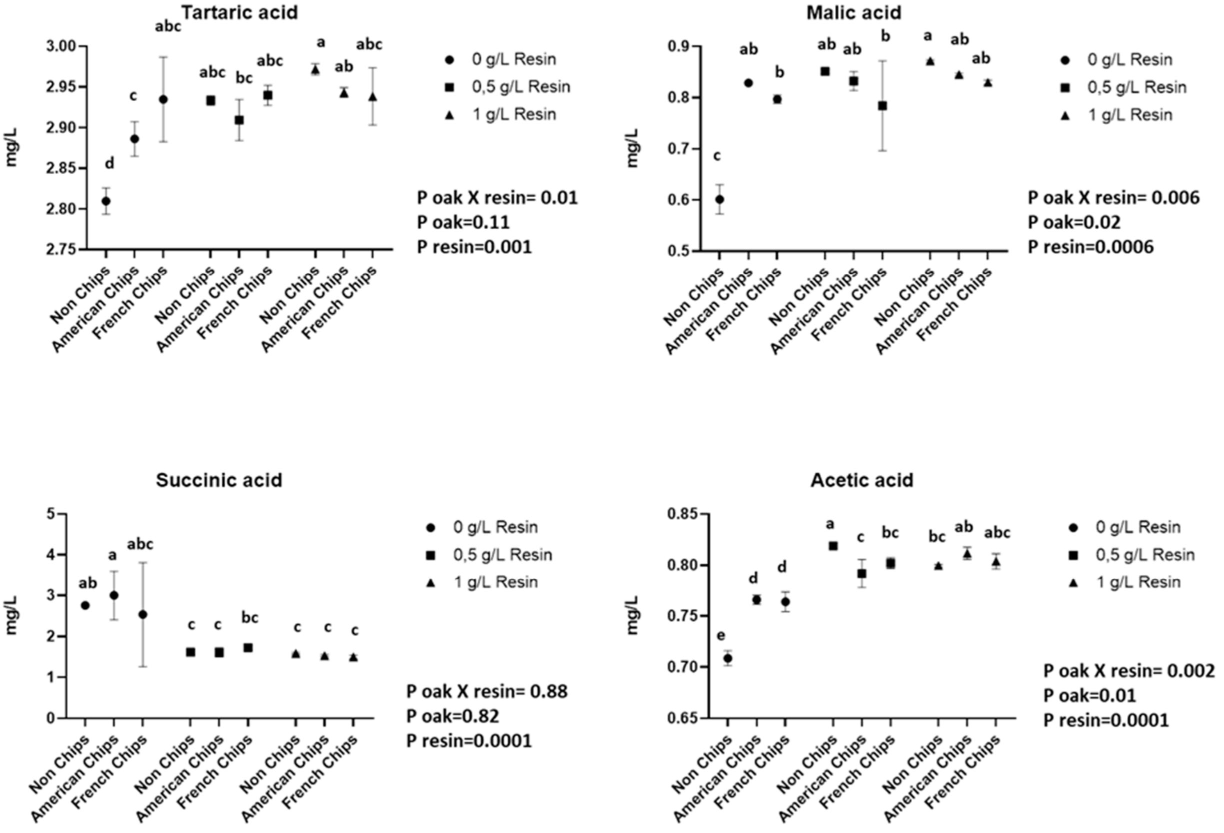

Figure S3. Plots presenting Organic acids of the experimental wines in response to two-way ANOVA, different resin treatments (0 g/L, 0.5 g/L and 1 g/L of resin) and different oak chips(no Chips, American oak and French oak), produced with the Vivace yeast. Data represent mean and standard deviations represented by error bars. P_{oak}, probability value for the addition of oak chips (American and French); P_{resin}, probability value for the resin (0.5 and 1 g/L) addition; $P_{oak \times resin}$, probability value for the oak × resin interaction. P values higher than 0.05 indicate lack of significant effect. Different letters between columns indicate significant differences at $p < 0.05$. (Tuckey multiple range test).

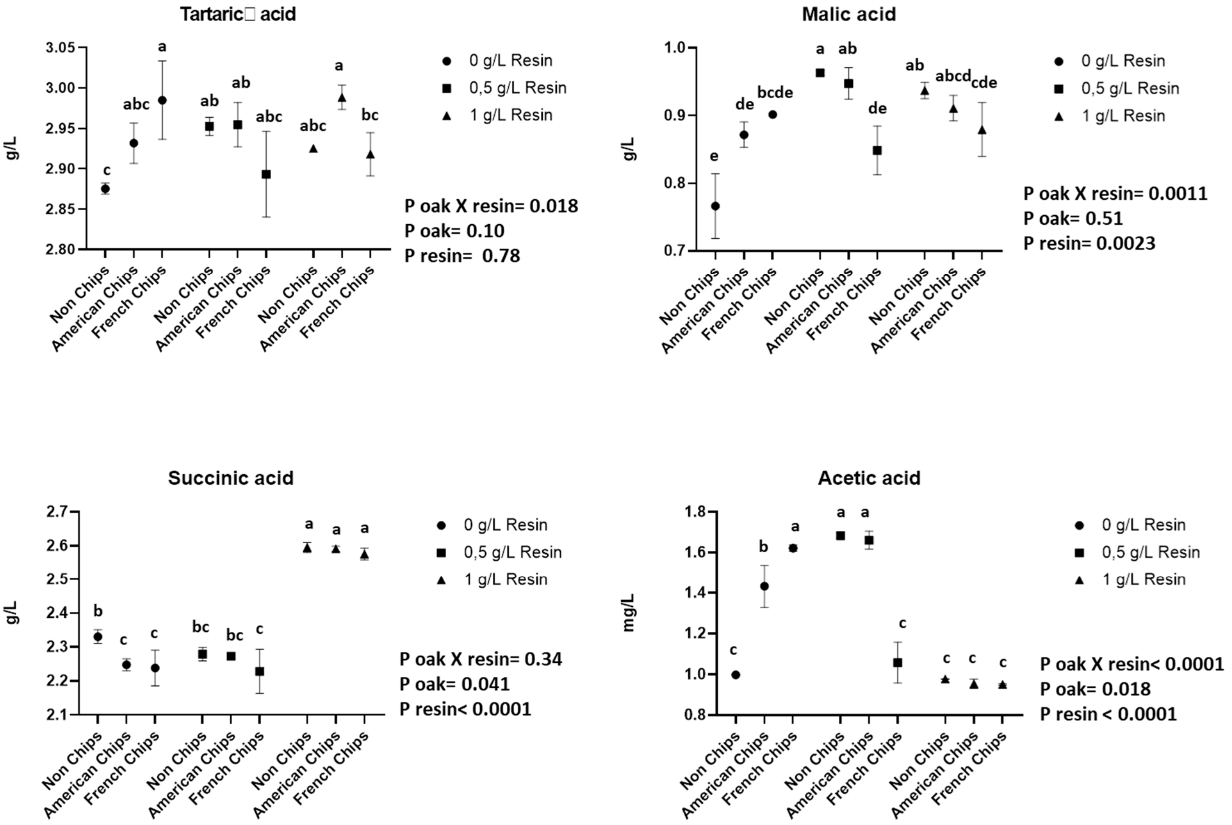

Figure S4. Plots presenting Organic acids of the experimental wines in response to two-way ANOVA, different resin treatments (0 g/L, 0.5 g/L and 1 g/L of resin) and different oak chips(no Chips, American oak and French oak), produced with the Zymaflore X5 yeast. Data represent mean and standard deviations represented by error bars. P_{oak}, probability value for the addition of oak chips (American and French); P_{resin}, probability value for the resin (0.5 and 1 g/L) addition; $P_{oak \times resin}$, probability value for the oak × resin interaction. P values higher than 0.05 indicate lack of significant effect. Different letters between columns indicate significant differences at $p < 0.05$. (Tuckey multiple range test).

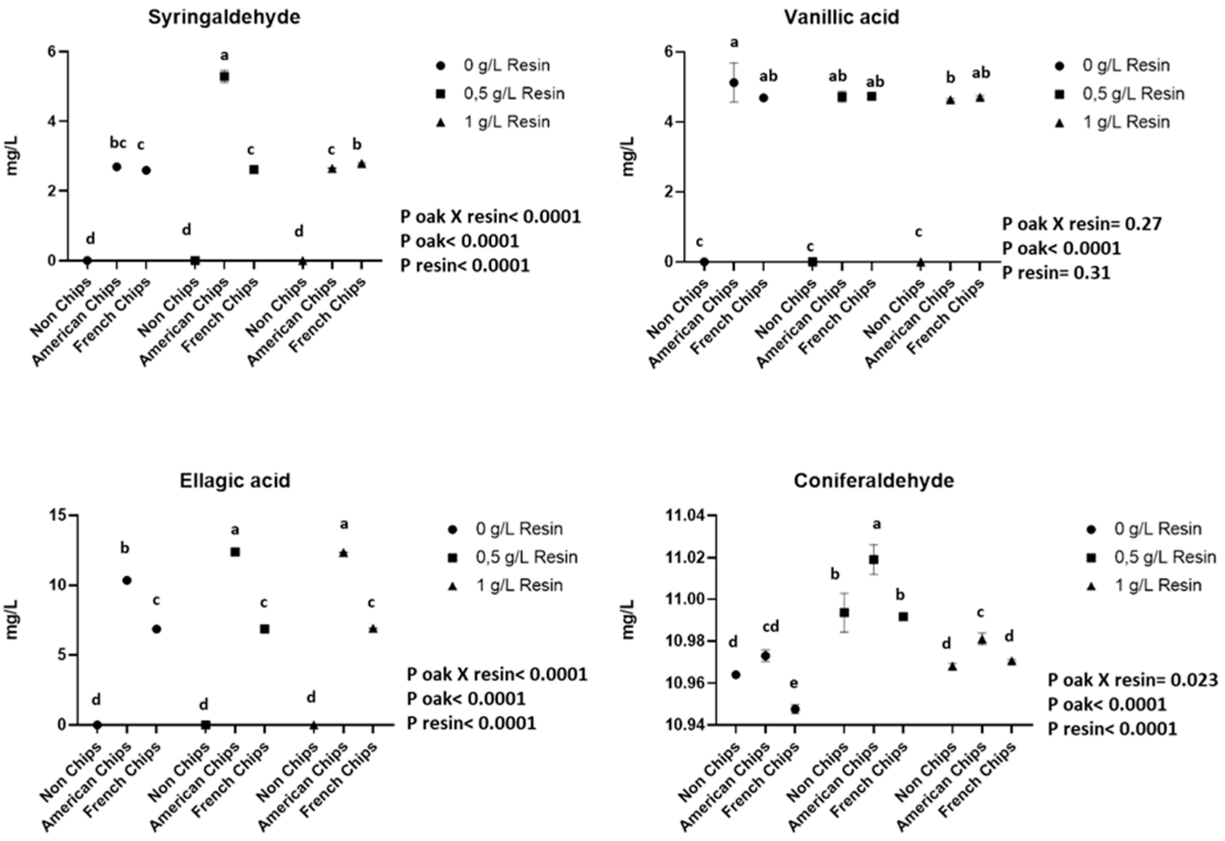

Figure S5. Plots presenting Phenolic compounds of the experimental wines in response to two-way ANOVA, different resin treatments (0 g/L, 0.5 g/L and 1 g/L of resin) and different oak chips(no Chips, American oak and French oak), produced with the Vivace yeast. Data represent mean and standard deviations represented by error bars. Poak, probability value for the addition of oak chips (American and French); P resin, probability value for the resin (0.5 and 1 g/L) addition; P oak x resin, probability value for the oak × resin interaction. P values higher than 0.05 indicate lack of significant effect. Different letters between columns indicate significant differences at $p < 0.05$. (Tuckey multiple range test).

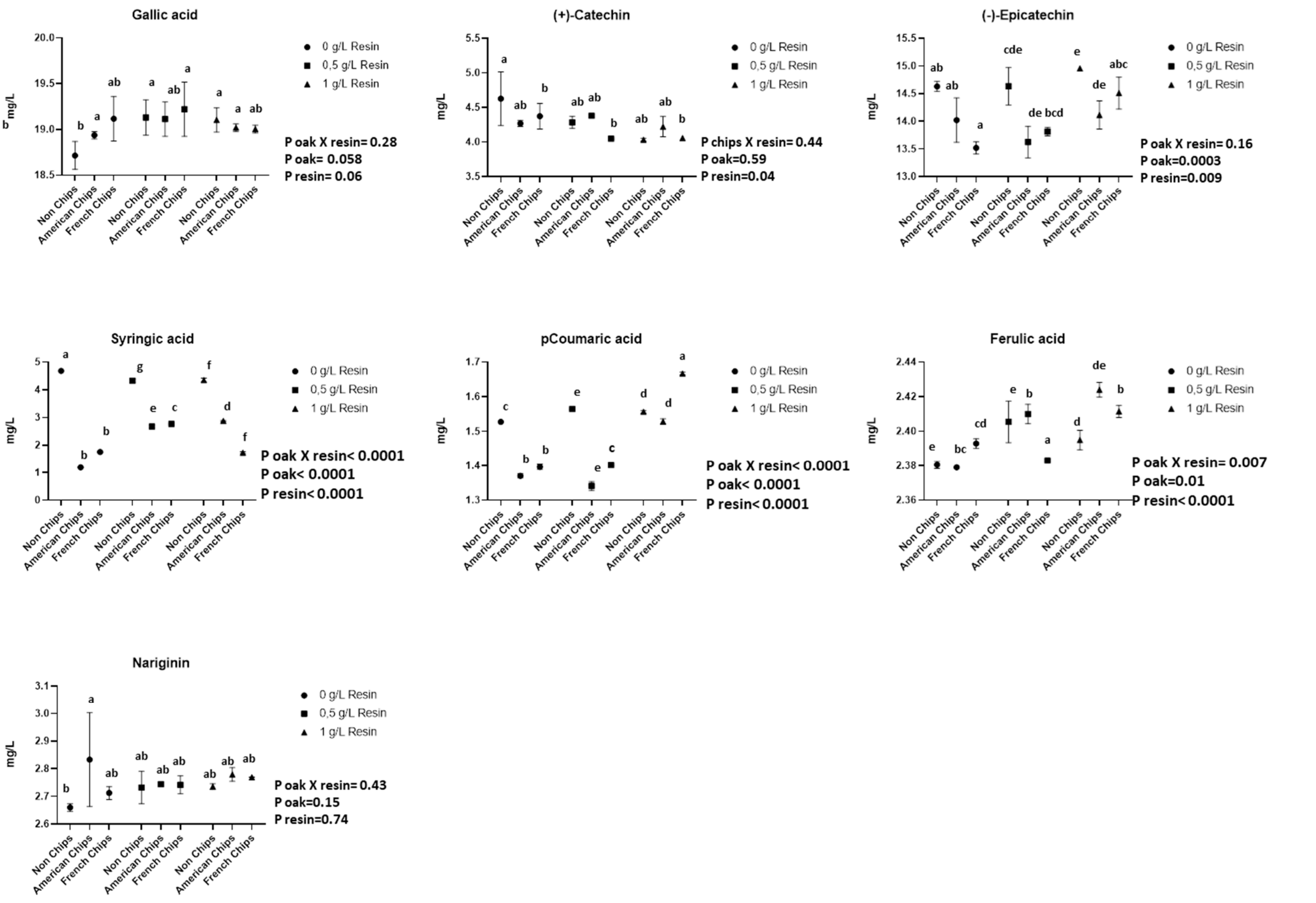

Figure S6. Plots presenting Phenolic compounds of the experimental wines in response to two-way ANOVA, different resin treatments (0 g/L, 0.5 g/L and 1 g/L of resin) and different oak chips(no Chips, American oak and French oak), produced with the Vivace yeast. Data represent mean and standard deviations represented by error bars. Poak, probability value for the addition of oak chips (American and French); P resin, probability value for the resin (0.5 and 1 g/L) addition; P oak x resin, probability value for the oak × resin interaction. P values higher than 0.05 indicate lack of significant effect. Different letters between columns indicate significant differences at $p < 0.05$. (Tuckey multiple range test).

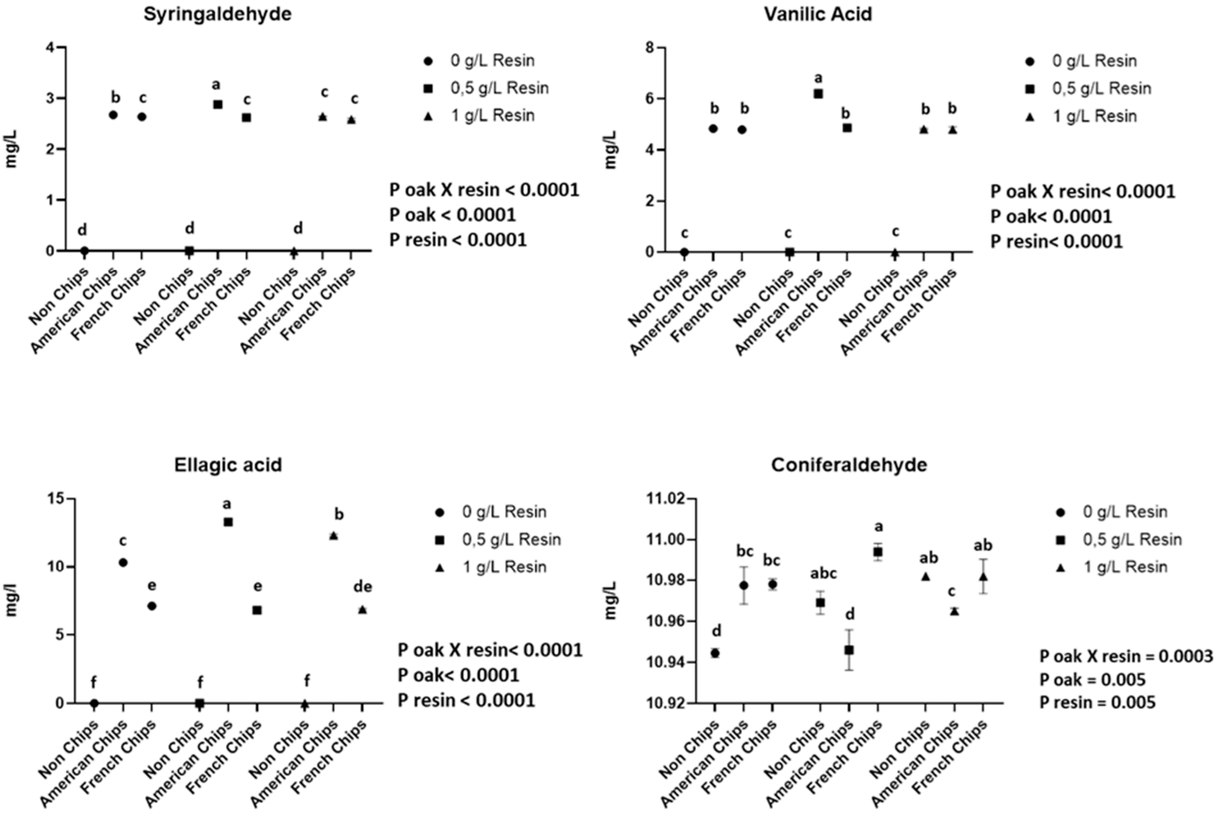

Figure S7. Plots presenting Phenolic compounds of the experimental wines in response to two-way ANOVA, different resin treatments (0 g/L, 0.5 g/L and 1 g/L of resin) and different oak chips(no Chips, American oak and French oak), produced with the Zymaflore X5 yeast. Data represent mean and standard deviations represented by error bars. Poak, probability value for the addition of oak chips (American and French); P resin, probability value for the resin (0.5 and 1 g/L) addition; P oak x resin, probability value for the oak × resin interaction. P values higher than 0.05 indicate lack of significant effect. Different letters between columns indicate significant differences at $p < 0.05$. (Tuckey multiple range test).

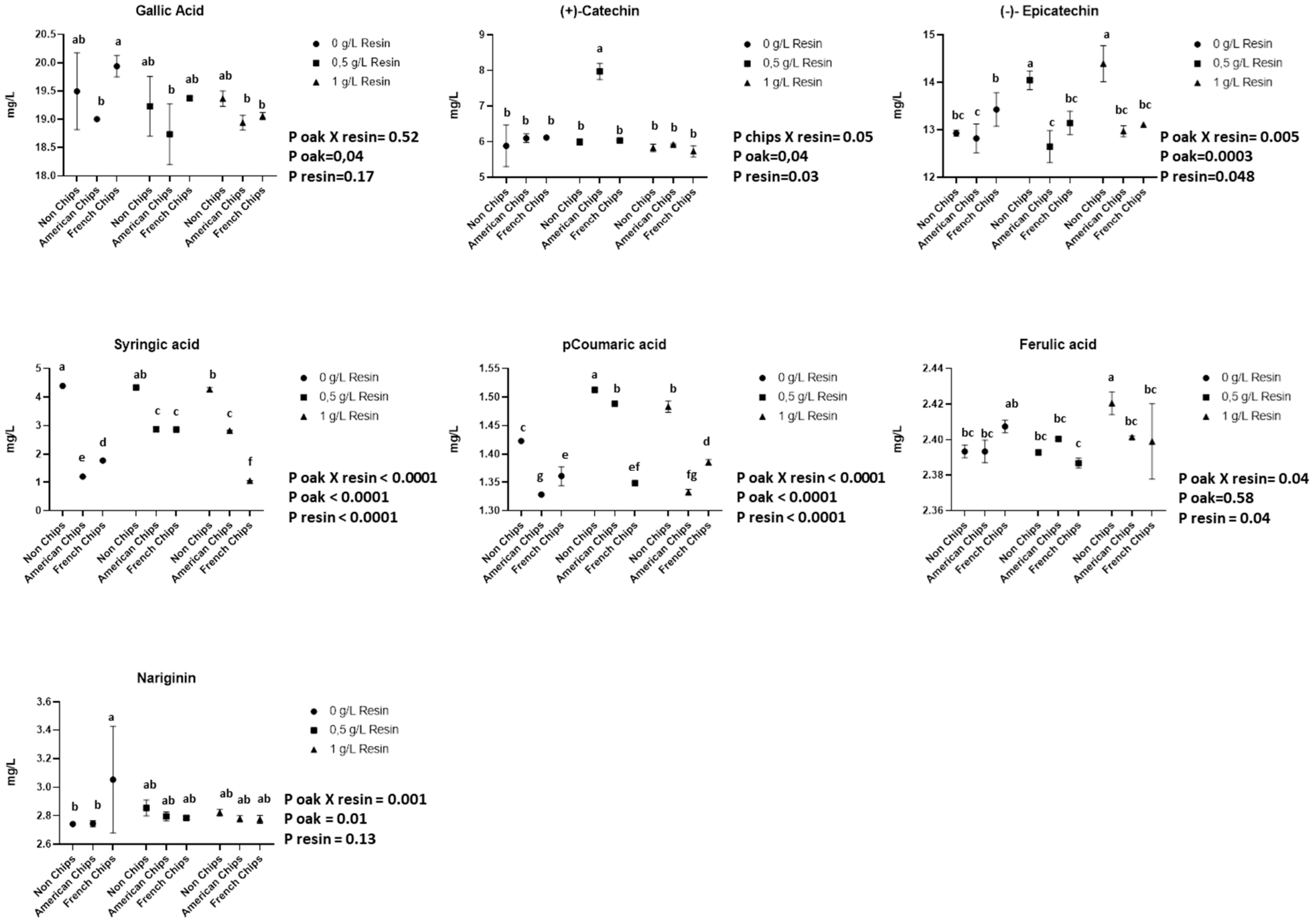

Figure S8. Plots presenting Phenolic compounds of the experimental wines in response to two-way ANOVA, different resin treatments (0 g/L, 0.5 g/L and 1 g/L of resin) and different oak chips(no Chips, American oak and French oak), produced with the ZYMAFLORE X5 yeast. Data represent mean and standard deviations represented by error bars. P_{oak}, probability value for the addition of oak chips (American and French); P resin, probability value for the resin (0.5 and 1 g/L) addition; P oak x resin, probability value for the oak × resin interaction. P values higher than 0.05 indicate lack of significant effect. Different letters between columns indicate significant differences at $p < 0.05$. (Tuckey multiple range test).

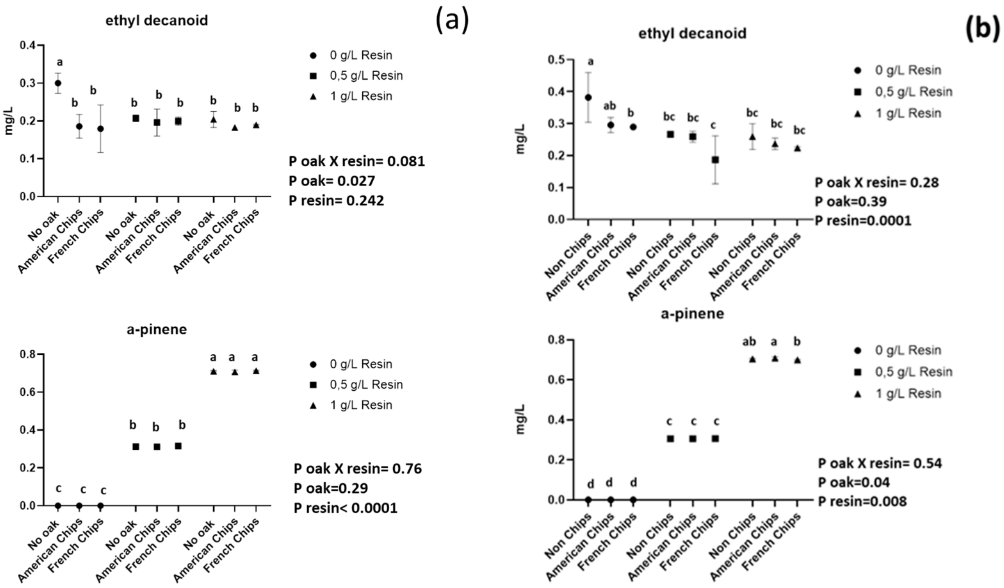

Figure S9. Plots presenting volatile compounds of the experimental wines Trend plots presenting Phenolic compounds of the experimental wines in response to two way ANOVA, different resin treatments (0 g/L, 0.5 g/L and 1 g/L of resin) and different oak chips(no Chips, American oak and French oak), produced with the Vivace (a) and Zymaflore X5 (b) yeast. Data represent mean and standard deviations represented by error bars. P_{oak}, probability value for the addition of oak chips (American and French); P_{resin}, probability value for the resin (0.5 and 1 g/L) addition ; $P_{oak \times resin}$, probability value for the oak × resin interaction. P values higher than 0.05 indicate lack of significant effect. Different letters between columns indicate significant differences at $p < 0.05$. (Tuckey multiple range test).

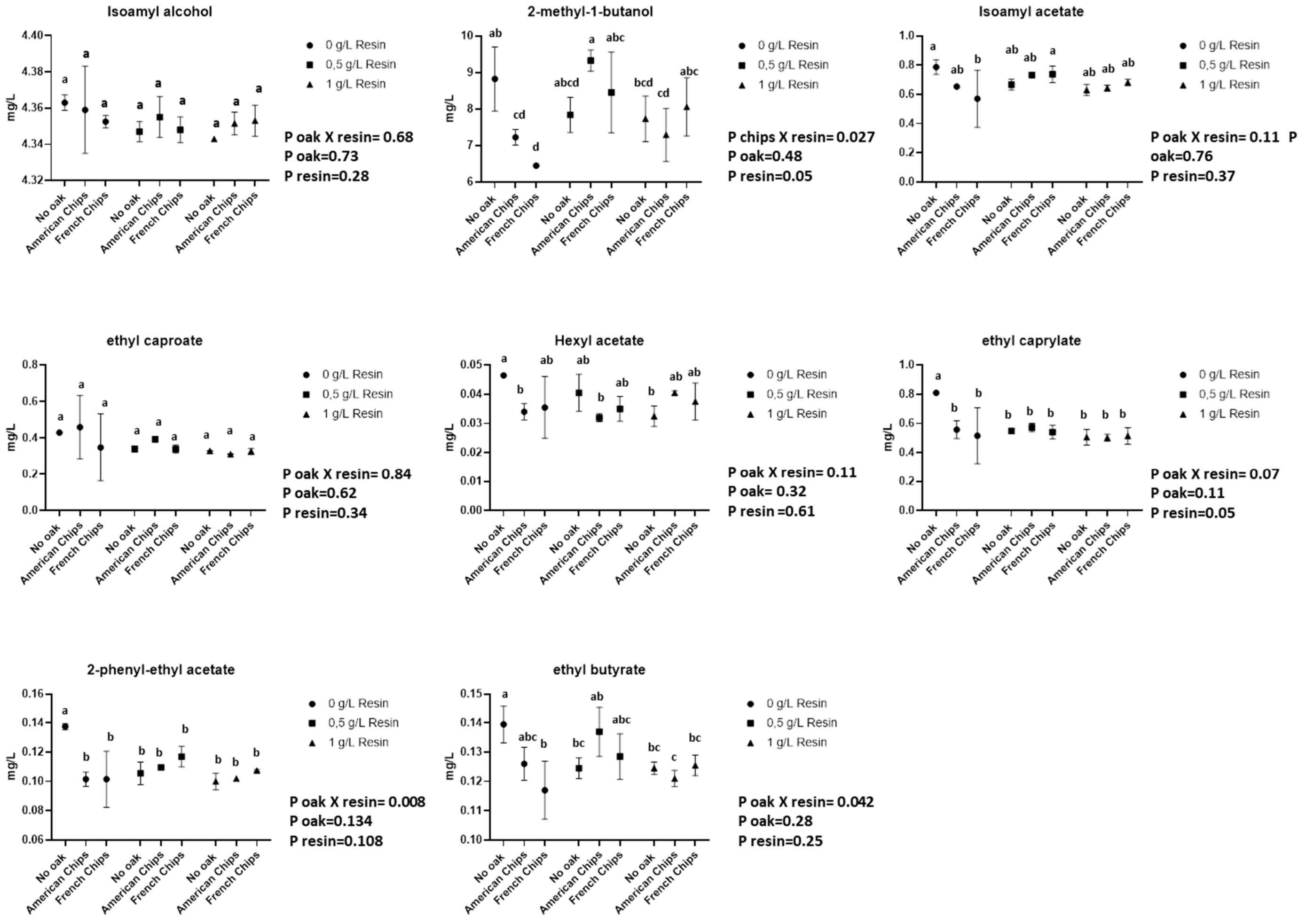

Figure S10. Plots presenting Volatile compounds of the experimental wines in response to two-way ANOVA, different resin treatments (0 g/L, 0.5 g/L and 1 g/L of resin) and different oak chips(no Chips, American oak and French oak), produced with the Vivace yeast. Data represent mean and standard deviations represented by error bars. P_{oak}, probability value for the addition of oak chips (American and French); P_{resin}, probability value for the resin (0.5 and 1 g/L) addition; $P_{oak \times resin}$, probability value for the oak × resin interaction. P values higher than 0.05 indicate lack of significant effect. Different letters between columns indicate significant differences at $p < 0.05$. (Tuckey multiple range test).

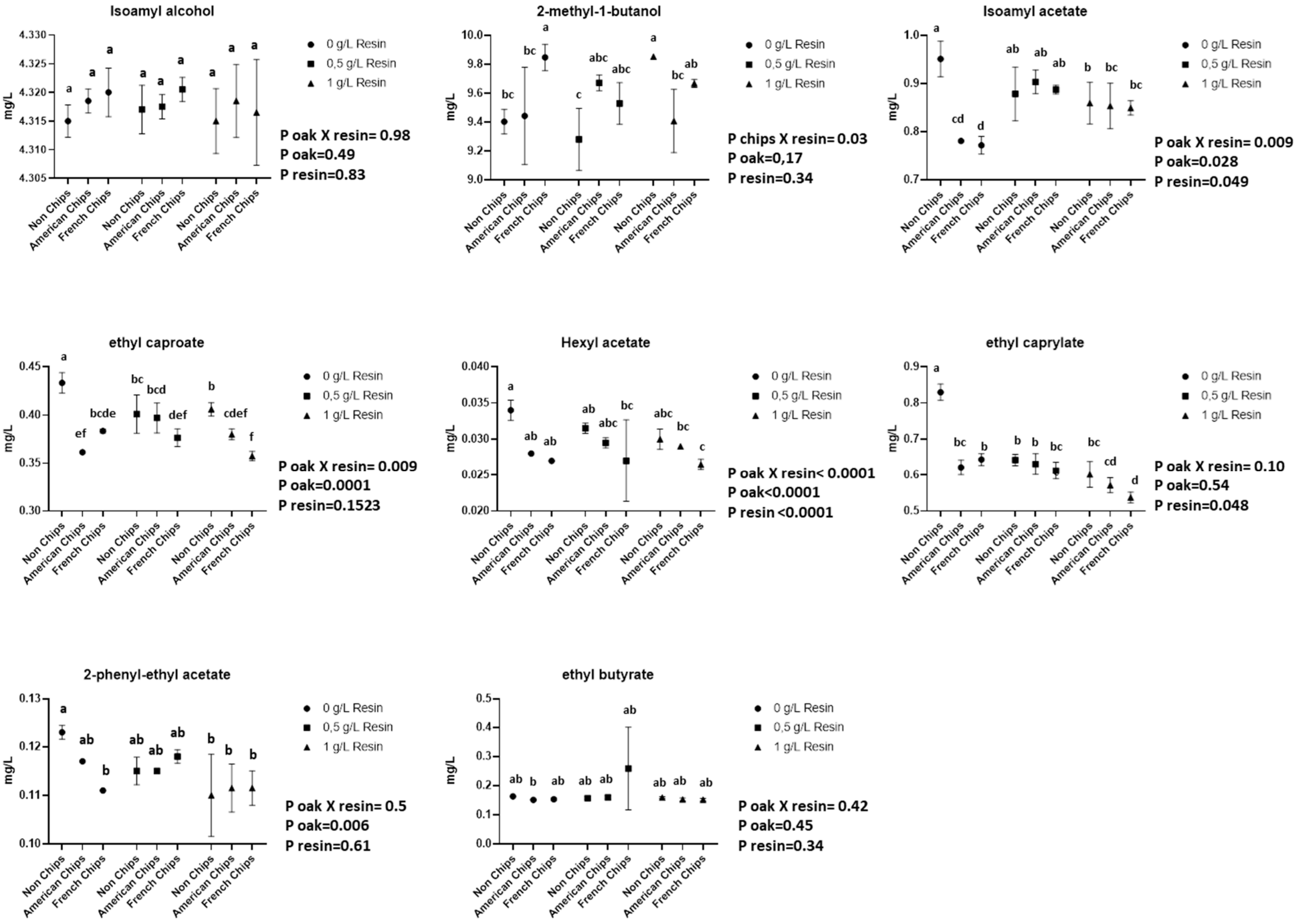

Figure S11. Plots presenting Volatile compounds of the experimental wines in response to two-way ANOVA, different resin treatments (0 g/L, 0.5 g/L and 1 g/L of resin) and different oak chips(no Chips, American oak and French oak), produced with the ZYMAFLORE X5 yeast. Data represent mean and standard deviations represented by error bars. P_{oak}, probability value for the addition of oak chips (American and French); P_{resin}, probability value for the resin (0.5 and 1 g/L) addition; $P_{oak \times resin}$, probability value for the oak × resin interaction. P values higher than 0.05 indicate lack of significant effect. Different letters between columns indicate significant differences at $p < 0.05$. (Tuckey multiple range test).

Table S1. Multiple analysis of variance to analyze the influence of the resin treatment and the oak chips level and their interaction in the aromatic series.

		Vivace			Zymaflore X5		
**Category descriptor**	**Descriptor**	**Oak**	**Resin**	**Interac-tion**	**Oak**	**Resin**	**Interac-tion**
Visual	**Colour Intensity**	ns	ns	ns	ns	ns	ns
Odor	**Aroma Intensity**	ns	ns	*	ns	***	***
Odor	**White Fruits**	ns	***	***	***	***	ns
Odor	**White Flowers**	*	***	***	***	***	***
Odor	**Vegetal/Grassy Aroma**	***	***	***	***	**	***
Odor	**Resin Aroma**	ns	***	***	ns	***	**
Odor	**Vanilla Aroma**	***	**	***	***	***	***
Odor	**Spiciness**	***	**	ns	***	***	***
Odor	**Woody Aroma**	***	**	**	***	***	***

Taste	**Acidity**	ns	ns	ns	ns	ns	ns
Taste	**Bitterness**	**	***	***	*	***	***
Taste	**Astringency**	***	***	ns	***	***	***
Taste	**Aftertaste**	ns	ns	ns	ns	***	ns

*p ≤ 0.05; **p ≤ 0.01; ***p ≤ 0.001; ns p ≥ 0.05; ns: not significant.

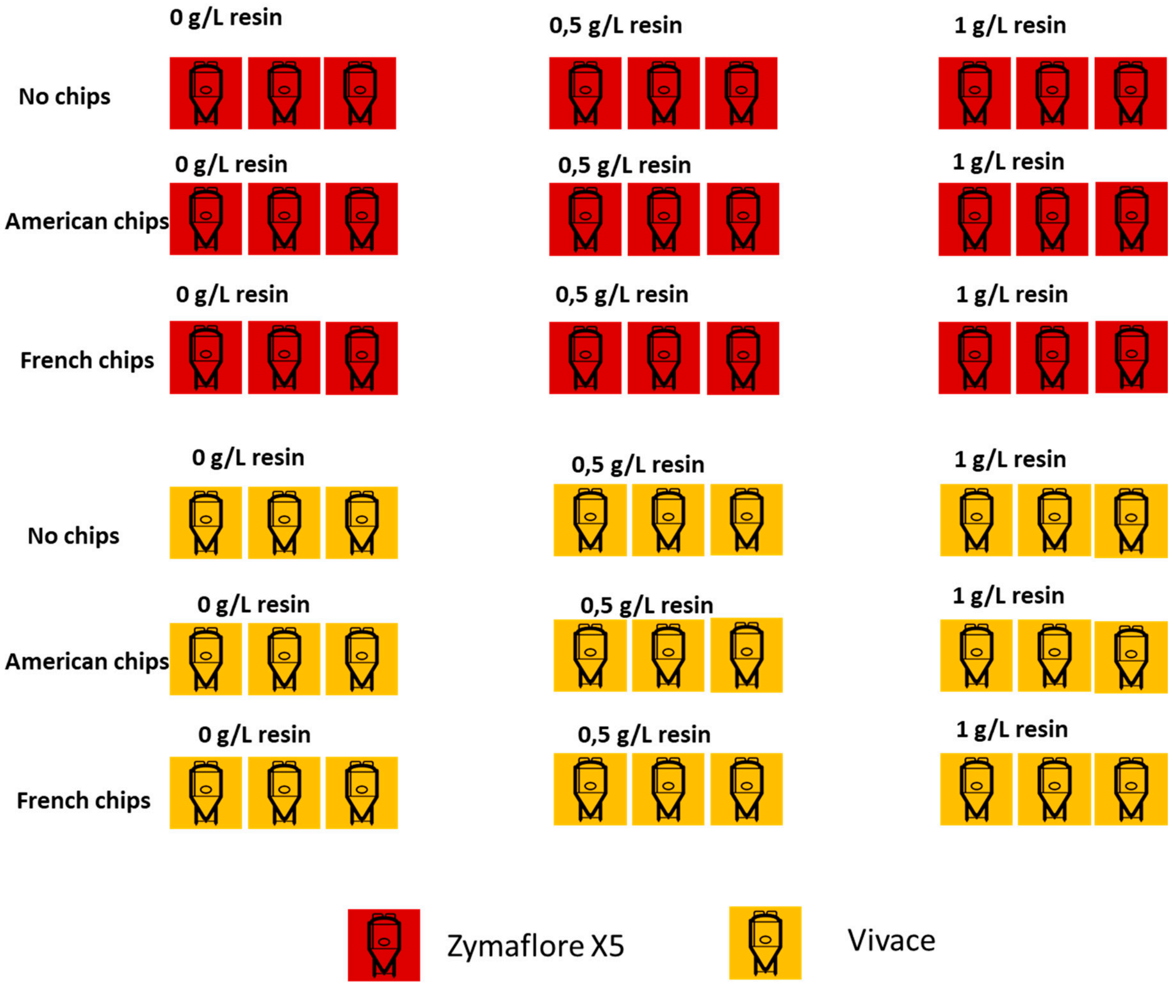

Figure S12. Experimental design for assessing the impact of resin concentration, oak chip type, and yeast strain on Retsina wine.
